# Modular type I polyketide synthase acyl carrier protein domains share a common N-terminally extended fold

**DOI:** 10.1038/s41598-019-38747-9

**Published:** 2019-02-20

**Authors:** Luisa Moretto, Rachel Heylen, Natalie Holroyd, Steven Vance, R. William Broadhurst

**Affiliations:** 1 Department of Chemistry and Biomedical Sciences, Linnaeus University, Smålandsgatan-24, 392 34 Kalmar, Sweden; 2 Department of Biochemistry, University of Cambridge, 80 Tennis Court Road, Cambridge, CB2 1GA UK; 3 Department of Medical Physics and Bioengineering, University College London, London, WC1E 6BT UK; 4 Crescendo Biologics Ltd, Meditrina Building 260, Babraham Research Campus, Cambridge, CB22 3AT UK

## Abstract

Acyl carrier protein (ACP) domains act as interaction hubs within modular polyketide synthase (PKS) systems, employing specific protein-protein interactions to present acyl substrates to a series of enzyme active sites. Many domains from the multimodular PKS that generates the toxin mycolactone display an unusually high degree of sequence similarity, implying that the few sites which vary may do so for functional reasons. When domain boundaries based on prior studies were used to prepare two isolated ACP segments from this system for studies of their interaction properties, one fragment adopted the expected tertiary structure, but the other failed to fold, despite sharing a sequence identity of 49%. Secondary structure prediction uncovered a previously undetected helical region (H0) that precedes the canonical helix-bundle ACP topology in both cases. This article reports the NMR solution structures of two N-terminally extended mycolactone mACP constructs, mH0ACPa and mH0ACPb, both of which possess an additional α-helix that behaves like a rigid component of the domain. The interactions of these species with a phosphopantetheinyl transferase and a ketoreductase domain are unaffected by the presence of H0, but a shorter construct that lacks the H0 region is shown to be substantially less thermostable than mH0ACPb. Bioinformatics analysis suggests that the extended H0-ACP motif is present in 98% of type I *cis*-acyltransferase PKS chain-extension modules. The polypeptide linker that connects an H0-ACP motif to the preceding domain must therefore be ~12 residues shorter than previously thought, imposing strict limits on ACP-mediated substrate delivery within and between PKS modules.

## Introduction

**Figure 1:**
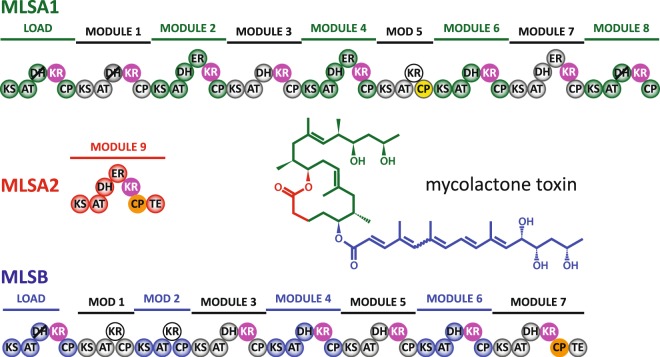
Module organization for the three subunits of the mycolactone PKS system (MLSA1, MLSA2 and MLSB)^7^. The structure of mycolactone is colour coded to match the subunits responsible for synthesizing each segment. mH0ACPa and mH0ACPb are shaded yellow and orange, respectively. A1- and B1-type KR domains are white and magenta, respectively. DH domains that are predicted to be inactive are marked with diagonal black lines. Domain abbreviations: KS, ketosynthase; AT, acyltransferase; KR, ketoreductase; DH, dehydratase; ER, enoyl reductase; CP, acyl carrier protein; TE, thioesterase.

The polyketide family of natural products is remarkable for both its structural diversity and biological activity, containing clinically important molecules such as macrolide, polyene and polyether antibiotics^1^. Polyketides are constructed from simple carboxylic acid-derived components, often by filamentous bacteria using modular type I polyketide synthases (PKSs), gigantic protein complexes that house multiple modules of covalently-linked catalytic domains^2^. Inside these systems, acyl carrier protein (ACP) domains act as key interaction hubs, shuttling substrate chains between the active sites in one module before handing their cargo over to the next. This role is facilitated by post-translational modification of the ACP at a conserved serine site with a 4′-phosphopantetheine (Ppant) prosthetic group derived from Coenzyme A (CoA)^3,4^. In *cis-*acyltransferase (AT) chain-extension modules, the AT domain recruits a building block (typically methylmalonyl-CoA) and loads its α-carboxyacyl moiety onto the Ppant arm of an ACP domain via a thioester linkage. The ACP delivers this extender unit to a ketosynthase (KS) domain for a decarboxylative condensation reaction that appends it to an acyl chain furnished by the previous module. Next, if tailoring domains are present, the ACP may visit ketoreductase (KR), dehydratase (DH) or enoylreductase (ER) active sites to reduce the β-ketone group of the substrate to an alcohol, eliminate the β-hydroxyl group to form an α-β double bond, or create a fully reduced β-methylene group, respectively. Finally, the ACP domain will either pass its substrate on to the KS domain of the next module or release it, for example through interaction with a C-terminal thioesterase (TE) domain. The multi-domain architectures of modular type I PKSs (Fig. 1) closely resemble those of iterative type I PKS and fatty acid synthase (FAS) complexes, and also those of *trans*-AT PKS systems, which lack integrated AT domains but are serviced by a free-standing AT that supplies extender units to each module^5^. Type II polyketide and fatty acid synthase systems, which consist of discrete domains, are more distantly related, but catalyse the same cycle of reactions^6^.

In *Mycobacterium ulcerans*, the toxin mycolactone is produced by a modular type I *cis-*AT PKS that comprises 16 chain-extension modules spread across three polypeptide chains, MLSA1, MLSA2 and MLSB (Fig. 1)^7^. This system employs two varieties of KR domain with characteristic amino acid sequence signatures, termed A1- and B1-type, which use an NADPH cofactor to generate substrate β-hydroxyl groups with 3 *S* and 3 *R* stereochemistry, respectively, but which leave the 2 *R* orientations of α-methyl substituents unchanged^8^. Our recent isothermal titration calorimetry (ITC) experiments demonstrated that interactions between either type of KR domain and an acyl-ACP species from the same module are multivalent, with contributions from the surface of the ACP domain, the prosthetic group and the thioester-linked substrate^9^. We have also used nuclear magnetic resonance (NMR) spectroscopy to show that the Ppant arm swings freely around its attachment point when loaded with short, polar substrate mimics, whereas longer, more saturated chains can adhere to the surface of the ACP domain without significantly altering its structure^10^.

The latter study focussed on mACP9, a polypeptide segment excised from MLSA2, which contains a B1-type KR domain and acts as the ninth chain-extension module of the PKS system that builds the mycolactone ring (Fig. 1); the amino acid sequence of mACP9 is identical to that of mACPb studied here. The boundaries of the mACP9 construct^10^ were chosen to match the structured core observed for *ery*ACP2, a fragment from the second chain-extension module of the PKS responsible for manufacturing 6-deoxyerythronolide B (6DEB), the aglycone core of the macrolide antibiotic erythromycin^11^. Here we report structural studies of an ACP domain from a mycolactone PKS module that contains an A1-type KR, demonstrating that a helical N-terminal extension (termed H0) is required for proper folding. In addition, the thermostability of the canonical ACP motif in our mACPb construct is shown to be enhanced by including an N-terminal H0 region. Sequence analysis indicates that the extended H0-ACP unit is a common feature in type I *cis*-AT PKS systems.

## Results

### ACP domain boundaries

Two constructs for the A1-type ACP domain from module 5 of MLSA1 were chosen for protein expression and preliminary characterization. For mACPa (spanning residues 11098–11185), the N-terminal boundary was selected based on the solution structure of mACPb, prior to the first expected helix (H1; predicted to start at 11100)^10^. A longer construct (mH0ACPa, residues 11087–11185) was designed to include a putative N-terminal helical region (H0; predicted to start at 11089). Both constructs were expressed fused to cleavable N-terminal His_6_-tags and were produced at >1 mg per litre of culture medium after initial purification by nickel affinity chromatography followed by incubation with proteinase. During size exclusion chromatography to separate the products, mACPa and mH0ACPa were found to elute differently, at 12.5 mL and 13.3 mL, corresponding to spherical species with apparent radii of 22 Å and 18 Å, respectively (Supplementary Fig. S4). In ^1^H nuclear magnetic resonance (NMR) spectra of *apo* mACPa (which lacks the Ppant prosthetic group), the backbone amide and methyl signals showed reduced chemical shift dispersion compared with spectra of mH0ACPa (Supplementary Fig. S5), consistent with the shorter construct being partially unfolded. By contrast, short (mACPb, residues 13784–13874) and N-terminally extended (mH0ACPb, residues 13774–13874) constructs for the B1-type ACP domain coded in module 7 of MLSB eluted at 13.7 mL and 13.6 mL, corresponding to species with apparent radii of 16 Å and 17 Å, respectively. The [^1^H,^15^N]-heteronuclear single quantum coherence (HSQC) spectra of the *apo* forms of mH0ACPa, mH0ACPb (Supplementary Fig. S6,S7) and mACPb^10^ displayed highly dispersed resonances, characteristic of proteins that possess tertiary structure.

### Solution structures of *apo* mH0ACPa and *apo* mH0ACPb

**Figure 2:**
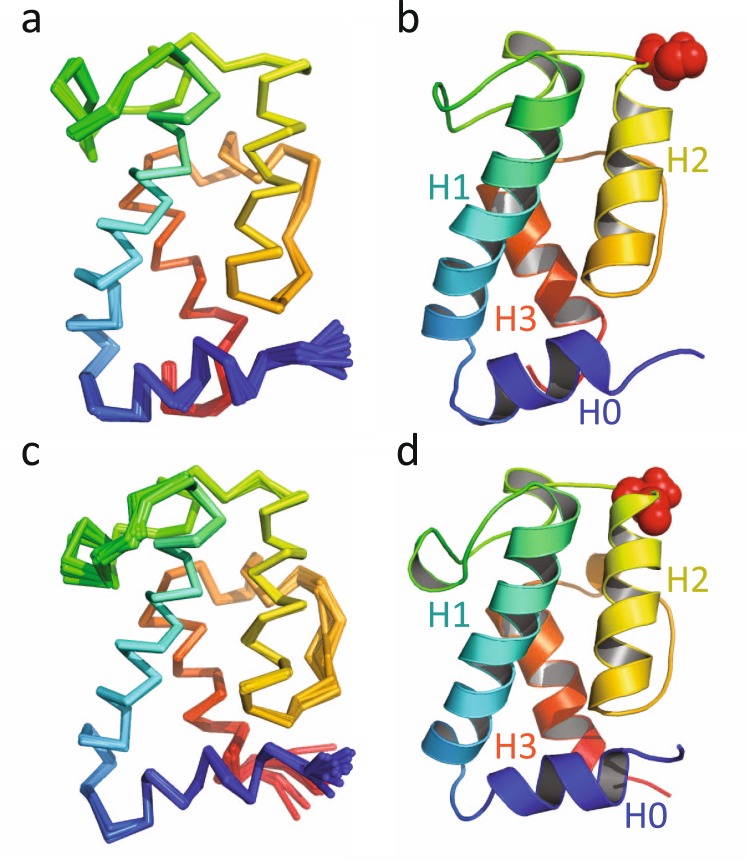
(**A**) Backbone overlay of ribbon representations for the final ensemble of structures for *apo* mH0ACPa, coloured from blue at the N-terminus to red at the C-terminus; and (**B**) cartoon representation of the lowest energy structure of mH0ACPa, with Ser11141 shown in red spheres. (**C**) Backbone overlay of ribbon representations for the final ensemble of structures for *apo* mH0ACPb, coloured from blue at the N-terminus to red at the C-terminus; and (**D**) cartoon representation of the lowest energy structure of mH0ACPb, with Ser13830 shown in red spheres.

A total of 2976 nuclear Overhauser effect (NOE)-derived distance restraints were used to determine the solution structure of the *apo* form of mH0ACPa. The final ensemble of structures was well-defined (Fig. 2A), with a backbone coordinate root mean square deviation (RMSD) of 0.1 Å over residues 11090 to 11184; further statistics are summarised in Table 1. mH0ACPa adopts the same right-handed twisted helical bundle (Fig. 2B) characterized previously in carrier proteins from PKS, FAS, and nonribosomal peptide synthetase (NRPS) systems^4^, with the novel addition of an extra α-helix at the N-terminus (H0, residues 11090–11096). Following this, three main α-helices (H1, 11101–11120; H2, 11143–11155; H3, 11170–11180) are interspersed with short helical turns (H2′ 11134–11137; and H3′ 11163–11166). H0 tucks into the base of the structure, opposite the face that displays Ser11141, the attachment point for Ppant modification. The side-chains of residues Leu11092 and Leu11096 (from H0) and Leu11099 (from the turn between H0 and H1) make non-polar contacts with Gln11104, Val11107, Thr11155, Ile11181 and Ile11184. The overall orientation of H0 with respect to the rest of the ACP domain is defined by 73 long range distance restraints between residues 11088–11099 and 11104–11184.

**Table 1:** Assignment, restraint and structure quality statistics for the solution structures of mH0ACPa and mH0ACPb.

Statistic description	mH0ACPa	mH0ACPb
Completeness of resonance assignments		
Backbone (%)	95.8	93.1
Aliphatic side-chain (%)	97.1	98.9
Aromatic side-chain (%)	100	100
NOE-based distance restraints		
Intra-residue	785	749
Sequential	690	458
Medium range	794	396
Long range	636	288
Semi-ambiguous	71	6
Total	2976	1897
Other restraints		
ϕ and ψ dihedral angle restraints	144	156
^1^H^N^-^15^N residual dipolar coupling restraints	75	67
Number of restraints per residue	32.0	20.8
Structures in ensemble/number refined in final step	20/35	15/35
Coordinate precision		
Backbone RMSD (Å)	0.14 ± 0.03	0.24 ± 0.07
Heavy atom RMSD (Å)	0.33 ± 0.06	0.50 ± 0.08
Consistency (structure vs restraints)		
RMSD (Å) from distance restraints	0.041 ± 0.007	0.053 ± 0.003
RMSD (°) from dihedral angle restraints	0.11 ± 0.08	0.07 ± 0.18
RMSD (Hz) from RDC restraints	0.88 ± 0.02	0.75 ± 0.04
PROCHECK Ramachandran plot summary^a^		
Most favoured regions (%)	87.8	85.3
Allowed regions (%)	12.2	11.6
Generously allowed regions (%)	0.0	0.1
Disallowed regions (%)	0.0	3.0
Global quality-scores^a^		
Verify 3D (mean score ± SD)	0.48 ± 0.01	0.42 ± 0.01
PROCHECK G-factor, ϕ – ψ (mean score)	−0.33	−0.29
PROCHECK G-factor, all (mean score)	−0.53	−0.46
MolProbity clash score (mean score ± SD)	65.4 ± 2.9	57.3 ± 1.9

The solution structure of the *apo* form of mH0ACPb was also calculated (Fig. 2C,D), with 1897 distance restraints yielding a backbone RMSD of 0.2 Å between residues 13777 and 13871 (Table 1). The H0 helix (residues 13778 to 13785) is again clearly defined, its orientation dictated by 40 long range distance restraints, with the side-chains of Leu13781, Leu13785 and Leu13788 making non-polar contacts with residues Gln13793, Thr13796, Leu13797, Thr13844 and Ile13870.

**Figure 3:**
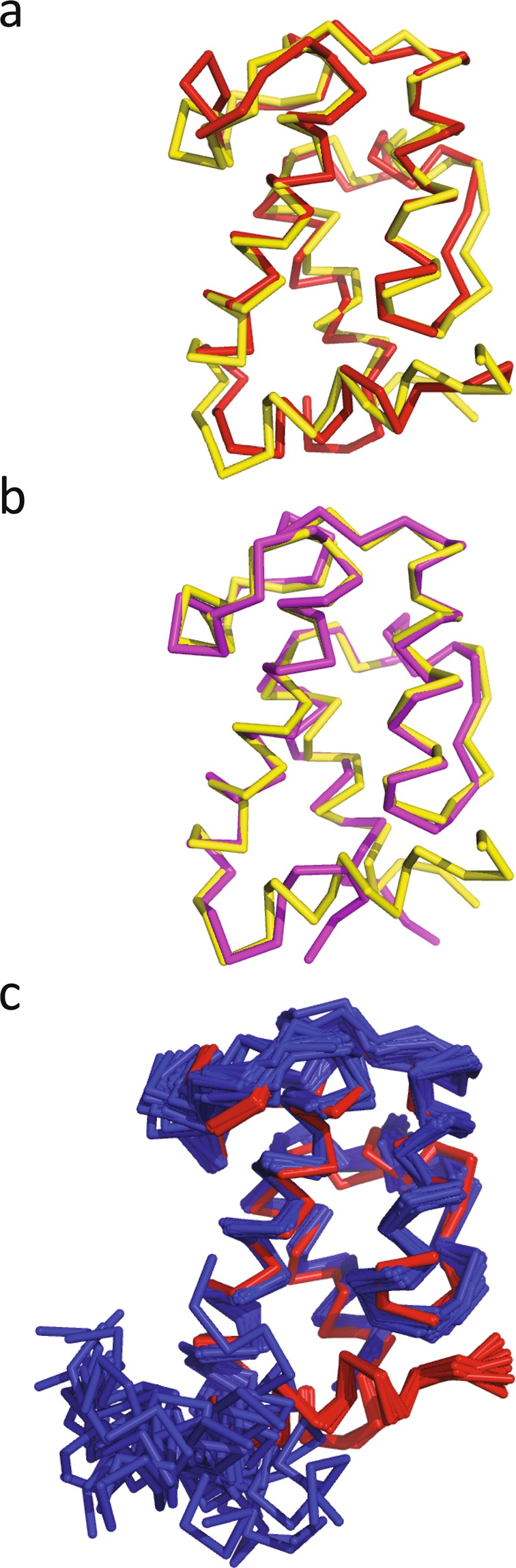
Superposition of ribbon representations of lowest energy solution structures for (**A**) *apo* mH0ACPa (red) and *apo* mH0ACPb (yellow); and (**B**) *apo* mACPb (magenta; PDB-ID 5HVC) and *apo* mH0ACPb (yellow). (**C**) Superposition of ensembles for *apo* mH0ACPa (red) and *apo* dACP2 (blue; PDB-ID 2JU1).

**Figure 4:**
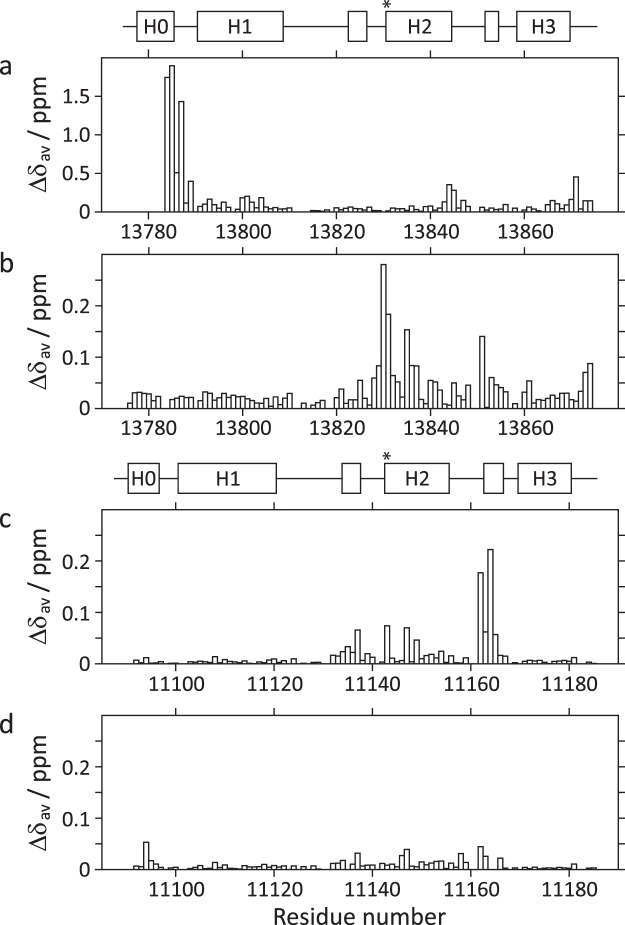
Average ^1^H^N^/^15^N chemical shift differences (Δδ_av_) plotted as a function of residue number, between: (**A**) *apo* mACPb and *apo* mH0ACPb; (**B**) *holo* mH0ACPb and *apo* mH0ACPb; (**C**) *holo* mH0ACPa and *apo* mH0ACPa; and (**D**) *holo* mH0ACPa and β-hydroxybutyryl-mH0ACPa. Schematics defining the boundaries of secondary structure elements in *apo* mH0ACPb and *apo* mH0ACPa are shown above panels (**A**) and (**C**), respectively, with asterisks indicating where the prosthetic groups are attached.

The amino acid sequences of the mH0ACPa and mH0ACPb constructs are 48% identical, so it is not surprising that their folds are closely related, showing a C^α^ RMSD of 1.1 Å between residues 11090 and 11181 (mH0ACPa numbering; Fig. 3A). The structure of mH0ACPb is also very similar to that of the previously studied mACP9 fragment^10^, which omitted 6 residues from the H0 region, showing a C^α^ RMSD of 0.7 Å from the start of helix H1 (residues 13784 to 13870, mH0ACPb numbering; Fig. 3B). When the [^1^H,^15^N]-HSQC spectra of mACPb and mH0ACPb are compared, large average ^1^H^N^/^15^N chemical shift differences are apparent for residues at the C-terminal end of H0, in the following turn and for sites towards the C-termini of H2 and H3, at the base of the domain (Fig. 4A). These changes are consistent with the local electronic environments of sensitive nuclei being altered by a loss of secondary structure in the truncated H0 sequence of mACPb.

The PDBeFOLD comparison server^12^ returns the same set of matches when probed with the structures of either mH0ACPa or mH0ACPb (Table 2). The top ranking hits are for ACP domains from modular type I PKS enzymes, the two highest being structures from the *trans*-AT system responsible for synthesizing the antibiotic virginiamycin^13^, VirA ACP5A (core RMSD, 1.5 Å to mH0ACPa; 1.4 Å to mH0ACPb) and VirA ACP5B (1.6 Å to mH0ACPa; 1.6 Å to mH0ACPb). These are followed by domains from the *cis*-AT systems that manufacture 6DEB (*ery*ACP2; 1.7 Å to mH0ACPa; 1.6 Å to mH0ACPb)^11^ and the anticancer agent curacin (CurAI; 1.7 Å to mH0ACPa; 1.6 Å to mH0ACPb)^14^. All four structures returned overlay the backbone heavy atoms of mH0ACPa and mH0ACPb closely from the start of H1 to the end of H3, but none match to the H0 region. Interestingly, the *ery*ACP2 construct omitted three residues that are predicted to initiate a H0 element; as a consequence, although 6 residues at the N-terminus of this DEBS1 fragment are arranged in a helical conformation, the helix adopts a wide variety of orientations in the solution structure ensemble (C^α^ RMSD 7.3 Å)^11^. Our structures for mH0ACPa and mH0ACPb behave differently, with the H0 helix consistently docking against the canonical ACP domain in a preferred conformation (RMSD 0.3 Å; Fig. 3C).

**Table 2:** Top ranked matches from the PDBeFOLD protein structure similarity server.

Category	VirA5A	VirA5B	*ery*ACP2	CurAI
PDB-ID	2MF4	4CA3	2JU1	2LIU
mH0ACPa				
Similarity Z-score	7.4	7.5	7.3	6.7
Core RMSD (Å)	1.5	1.6	1.7	1.7
Aligned residues	79	77	83	79
Sequence identity (%)	32	22	41	22
mH0ACPb				
Similarity Z-score	8.7	8.4	8.4	6.7
Core RMSD (Å)	1.4	1.6	1.6	1.6
Aligned residues	80	78	80	82
Sequence identity (%)	29	26	42	23

### Nuclear spin relaxation studies

**Figure 5:**
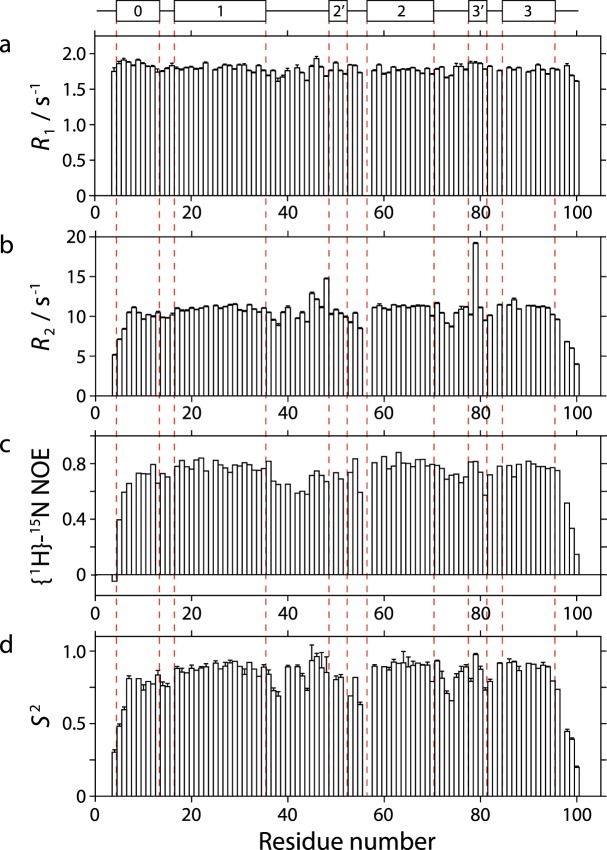
Underneath a schematic defining the boundaries of the secondary structure elements in *apo* mH0ACPa, nuclear spin relaxation parameters for backbone amide sites are plotted as a function of residue number for: (**A**) the ^15^N longitudinal relaxation rate, *R*_1_; (**B**) the ^15^N transverse relaxation rate, *R*_2_; (**C**) the {^1^H}-^15^N nuclear Overhauser effect ratio (I’/I_0_, where I’ is the intensity when the ^1^H spectrum has been saturated and I_0_ is the intensity in the reference spectrum); and (**D**) the Lipari-Szabo the order parameter, *S*^2^.

To investigate further whether the H0 region behaves as part of the ACP domain or shows signs of independent motion, nuclear spin relaxation parameters were measured for ^15^N-labelled amide sites in the *apo* form of mH0ACPa (Fig. 5). The data fitted best to an axially symmetric model of the rotational diffusion tensor, with a *D*_par_/*D*_perp_ ratio of 1.2 and an overall rotational correlation time of 8.4 ns, consistent with expectations for a monomeric, globular 100 amino acid domain in aqueous solution at 298 K^15^. Backbone amide sites were found to be predominantly rigid between Leu11092 and Ile11181, displaying a mean order parameter *S*^2^ of 0.88 ± 0.05. The mean *S*^2^ value for sites in H0 is 0.80 ± 0.14, compared with 0.90 ± 0.02 for sites in H1, H2 and H3; this minor discrepancy is likely due to dynamic fraying^16^ for two sites at the N-terminal end of the seven residue H0 helix. Lower values for the transverse relaxation rate *R*_2_ and the steady state {^1^H}-^15^N heteronuclear Overhauser effect ratio provide evidence for the influence of sub-nanosecond timescale motions on the relaxation properties of sites in the loops between H1 and H2′ and between H2 and H3′ (Fig. 5). Residues Ser11133 and Ile11164 exhibit enhanced transverse relaxation, consistent with conformational exchange processes occurring on the millisecond timescale and requiring *R*_ex_ contributions of 4.1 s^−1^ and 7.1 s^−1^, respectively. The overall picture for mH0ACPb is very similar, with mean order parameter values of 0.76 ± 0.13 and 0.85 ± 0.06 for sites in H0 and in H1, H2 and H3, respectively; these experiments were collected at a lower temperature (283 K) to minimize cross-peak overlap, resulting in a longer global correlation time of 9.4 ns, alongside the same *D*_par_/*D*_perp_ ratio of 1.2 (Supplementary Fig. S8).

### Thermostability experiments

The results described above suggest that the *apo* form of the shorter mACPa construct may have failed to fold because it lacked crucial stabilizing interactions with residues in the H0 region. This hypothesis was tested by using circular dichroism spectroscopy to monitor the thermal denaturation of two more stable species: the *apo* forms of mACPb and mH0ACPb (Supplementary Fig. S9). Both samples exhibited monophasic unfolding transitions, characterized by melting temperatures of 327 K and 338 K for mACPb and mH0ACPb, respectively. The shape of the profile observed for mH0ACPb is that expected for a two-state unfolding transition, implying that the H0 and canonical ACP regions behave as part of the same unit of tertiary structure, unravelling cooperatively rather than sequentially. The observed 11 K increase in melting temperature confirms that, when present, residues in the H0 region make a significant contribution to the stability of the folded state for mycolactone PKS carrier protein domains.

### Intermolecular and intramolecular interactions

Attachment of Ppant to the serine residue located at the start of H2 is mandatory for the activation an ACP domain^3,4^. Uniformly ^15^N-labelled *holo* forms of mH0ACPa and mH0ACPb were prepared by co-expression with Sfp (Supplementary Fig. S3), confirming that both N-terminally extended ACP species are recognized as substrates by this broad-specificity phosphopantetheinyl transferase. Backbone assignments for the *apo* forms were transferred to [^1^H,^15^N]-HSQC spectra of the corresponding *holo* species and verified using NOE connections. Average ^1^H^N^/^15^N chemical shift difference (Δδ_av_) profiles (Fig. 4B,C) demonstrate that the majority of backbone amide sites experience only minor perturbations in their electronic environment (<0.05 ppm), suggesting that no major structural changes occur when the *apo* ACP species are transformed into their *holo* forms. For both domains, moderate differences are observed for residues adjacent to the attachment site (mH0ACPa, 11141S; mH0ACPb, 13830S) and for residues towards the N-terminus of the nearby α3′ turn, which are contacted intermittently by the highly flexible Ppant prosthetic group^10^.

Acyl-ACP derivatives can be prepared by *in vitro* incubation of *apo* species with acyl-CoA thioesters in the presence of recombinant Sfp. Having previously used mACP9 derivatives to investigate the consequences of loading a panel of acyl substrate mimics^10^, here we focussed on β-hydroxybutyryl-mH0ACPa. Linkage of this short, polar C_4_-chain to the end of the Ppant group had little effect on cross-peak positions or intensities in comparison with the [^1^H,^15^N]-HSQC spectrum of *holo* mH0ACPa (Fig. 4D), implying that the modification introduced no preferential interactions with patches on the surface of the carrier protein.

**Figure 6:**
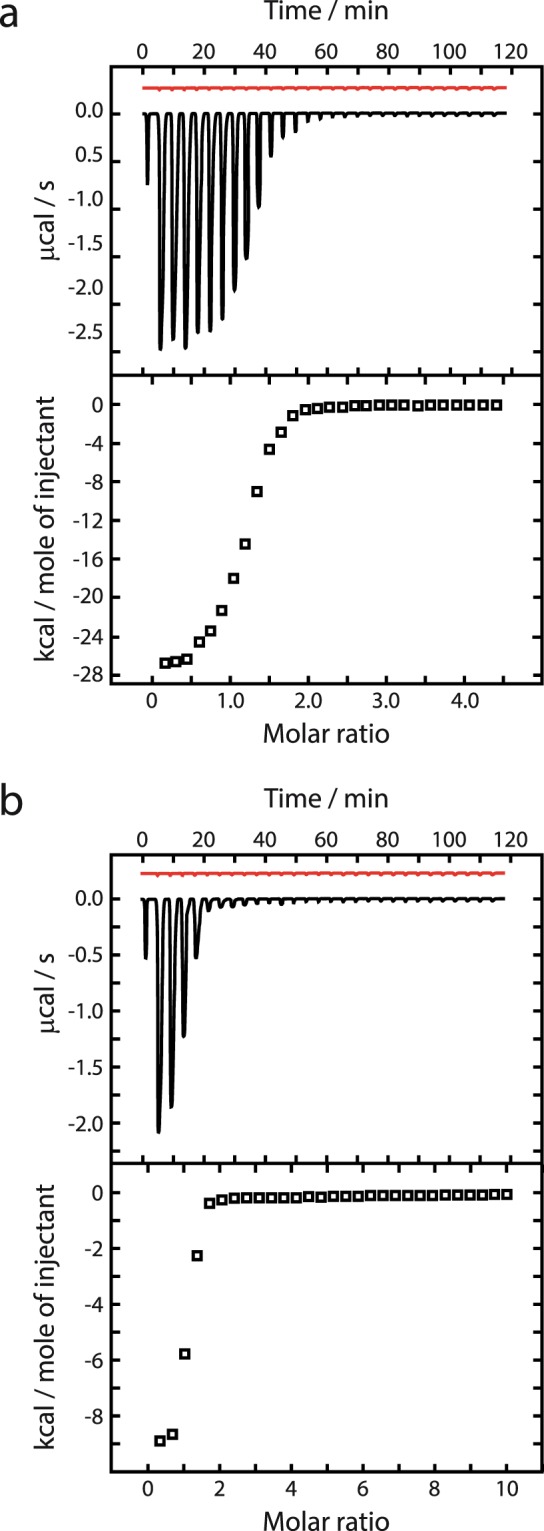
Representative ITC thermograms (upper panels) and isotherm plots (lower panels), showing consecutive injections of (**A**) *apo* mH0ACPa and (**B**) *holo* mH0ACPa, both against mKRb in the presence of NADPH. Thermogram traces for dilution control experiments are displayed in the upper panels in red.

To discover whether inclusion of the H0 region might affect the encounter between an ACP and a partner enzyme from a type I PKS module, we expressed and purified a recombinant fragment spanning the ketoreductase domain from module 7 of MLSB (mKRb)^9^. In an isothermal titration calorimeter, serial injection of *apo* mACPb into a solution containing mKRb and its cofactor NADPH produced a sequence of exothermic heat changes, confirming that this pair of domains excised from the same module can form a stable protein complex (Supplementary Fig. S10). Integration of the resulting thermogram yielded a sigmoidal isotherm plot consistent with 1:1 binding stoichiometry and a dissociation constant (*K*_D_) of 2.2 μM (Table 3). Replacing NADPH with NADP^+^ had no significant effect on the affinity of *apo* mACPb for mKRb (Supplementary Fig. S11), indicating that the interface between the two protein partners is not perturbed by the redox state of the cofactor^9^. Substitution of *apo* mACPb with the longer construct mH0ACPb (Table 3) also had similar consequences (*K*_D_ 1.4 μM), suggesting that the presence or absence of a H0 region has minimal impact on the interaction with a KR domain. By contrast, covalent attachment of Ppant to either ACP domain increased their affinities for mKRb to 0.7 μM (Table 3), demonstrating that the prosthetic group is likely to play a meaningful role in extending the interface between the ACP domain and the KR/cofactor binary complex^9^. Finally, titration of the non-cognate domain mH0ACPa (from module 5 of MLSA1) against mKRb achieved comparable results to the cognate species, independent of whether or not the Ppant group was attached (Fig. 6). This observation implies that the KR/cofactor complex must possess an interface that recognizes features common to all three investigated ACP constructs (mH0ACPa, mH0ACPb and mACPb).

**Table 3:** Dissociation constants, *K*_D_, for interactions between mKRb and various ACP species.

	mKRb + NADPH/μM	mKRb + NADP^+^/μM
*apo* mH0ACPa	2.20 ± 0.55	—
*apo* mACPb	2.20 ± 0.62	2.60 ± 0.40
*apo* mH0ACPb	1.43 ± 0.44	1.38 ± 0.29
*holo* mH0ACPa	0.63 ± 0.05	—
*holo* mACPb	0.75 ± 0.11	0.61 ± 0.23
*holo* mH0ACPb	0.69 ± 0.07	0.71 ± 0.05

### Presence of the H0 region in other megaenzyme systems

To investigate the prevalence of the N-terminal extension to the ACP domain provided by the H0 region, the SSpro secondary structure prediction server^17^ was used to analyse the primary sequence environment of 770 carrier protein domains. H0 extensions were predicted to be ubiquitous in *cis*-AT modular type I PKS systems, preceding canonical ACP motifs in 73% of loading modules and 98% of chain-extension modules (Table 4), including the second chain extension module of the 6DEB PKS (i.e. prior to *ery*ACP2). Loading module composition appears to correlate closely with the presence of a H0 region, from unlikely if the ACP under scrutiny is preceded directly by another ACP (e.g. in the curacin PKS system) or by none of the usual domains (24%, *n* = 17), to more frequent in AT-ACP didomain modules (67%, *n* = 6), to being the norm in configurations that contain KS and at least one other enzyme domain (96%, *n* = 23). Helical N-terminal extensions are less common for ACP domains in iterative type I PKS (31%) and modular NRPS/PKS hybrid systems (26%). H0 regions rarely feature in type I fatty acid synthase (0%) or *trans*-AT modular type I PKS systems (3%), such as the virginiamycin synthase, and are also not normally found upstream of canonical peptidyl carrier protein domains from NRPS systems (2%). This uneven distribution of H0 motifs (Table 4) probably reflects the separate evolutionary trajectories of different megaenzyme families^6^.

**Table 4:** Predicted presence of H0 regions in ACP domains from modular synthase systems.

Type	Chain role of module	Number	% predicted to possess H0
Type I modular PKS (*cis*-AT)	All	518	96
Type I modular PKS (*cis*-AT)	Loading	52	73
Type I modular PKS (*cis*-AT)	Extending	466	98
Type I iterative PKS (*cis-*AT)	Loading/extending	16	31
Type I modular PKS (*trans*-AT)	All	106	3
Type I hybrid NRPS/PKS	All	57	26
Type I hybrid NRPS/PKS	All	67	2
Type I FAS (*cis*-AT)	Loading/extending	6	0

When present in an intact modular type I PKS polypeptide chain, the H0 region is predicted to contain a helix with a mean length of 8 residues that is moored to the subsequent H1 helix by a 4 residue turn (Table 5). The unstructured linker sequence that connects each H0-ACP unit to the preceding domain tends to be longer in loading modules (19 residues) than in chain extension modules (10 residues). Modules in which the H0-ACP unit follows directly after an AT domain are also typically associated with pre-H0 linkers that are longer (25 residues) than those in which it appears after a KR domain (8.5 residues).

**Table 5:** Predicted lengths of helices and linkers in *cis*-AT modular type I PKS systems.

Species	Mean length/aa	Stdev	*n*
H0 length	7.5	1.5	495
Turn between H0 and H1	3.7	0.6	495
Loading module pre-H0 linker	18.6	13.4	53
Extension module pre-H0 linker	9.6	6.3	465
KR-ACP pre-H0 linker	8.5	4.0	416
AT-ACP pre-H0 linker	25.0	11.4	47

Alignments focussed on the H0 region reveal a conserved LAARLAGL sequence motif, where “L” sites are most often leucine, “A” sites are usually occupied by alanine, arginine or glutamate, the “R” site is polar and often positively charged, and the “G” site is enriched in residues with low volume side-chains (such as alanine, glycine or serine; Supplementary Fig. S12A). This sequence pattern is consistent with an amphipathic helix that displays a non-polar surface suitable for docking into the base of the ACP domain, followed by a tight turn that buries the side-chain of the third “L” site. In addition, evolutionary covariance analysis of a multiple sequence alignment of ACP domains from type I PKS systems predicts close contacts across the hinge between H0 and H1, connecting residues Lys11095 to Val11107, Leu11096 to Gln11104 and Leu11099 to Val11107 (Supplementary Fig. S13).

## Discussion

In this investigation we used solution-state NMR spectroscopy to characterize the structures of two ACP domains, mH0ACPa and mH0ACPb, excised from mycolactone PKS chain-extension modules that contain A1- and B1-type KR domains, respectively. The structures are highly similar to those of previously studied carrier proteins, in particular for ACPs from modular type I PKS systems^10,11,13,14^, apart from the presence of H0, an additional N-terminal α-helix that nuclear spin relaxation experiments confirm behaves like a rigid component of the tertiary structure of each domain. CD-monitored denaturation assays indicate that the mH0ACPb construct is substantially more thermostable than the N-terminally truncated (but still well-folded) mACPb fragment. Together with size exclusion chromatography and NMR data consistent with the N-terminally truncated mACPa construct being unfolded, this result implies that the overlooked H0 region may play a significant role in maintaining the functionality of carrier protein domains in megasynthase systems.

Even when experimental atomic resolution structures are available, the process of defining the boundaries of a protein fragment that can fold independently remains ambiguous, with algorithms based on dynamic, evolutionary, functional, geometric or thermodynamic definitions of a domain yielding conflicting results^18^. The task is more difficult still if the boundaries must be parsed from sequence information alone, for example when designing expression constructs for portions of a multi-domain protein to facilitate structural studies. Pragmatic approaches rely on intrinsic disorder and secondary structure prediction^19^ alongside comparisons with homologues of known structure to estimate where each domain begins and ends. Success is not guaranteed and minor adjustments can have sizeable effects, such as the 11 kJ mol^−1^ increase in stability obtained when a fragment from human tenascin was C-terminally extended by just two residues^20^. High-throughput structural genomics programs address this issue by assessing the solubility and expression level of multiple protein constructs with N- and C-terminal extensions or deletions, sometimes including fluorescent C-terminal fusion tags to aid detection^21^. Previous studies of modular type I PKS ACP domains^10,11^ were no doubt influenced by sequence similarity to shorter standalone proteins from type II systems; for example, the current PFAM template for a Ppant-binding domain is only 67 amino acids long^22^, compared with the 94-residue structured cores defined here for mH0ACPa and mH0ACPb. In light of the stability of some N-terminally truncated fragments, such as *ery*ACP2^11^, and the relative simplicity of the LAARLAGL sequence, it is perhaps not surprising that the importance of the H0 region should only now have become apparent.

The 11 K increase in melting temperature observed on inclusion of the H0 region prior to the canonical mACPb construct is similar in magnitude to the ~10 K stabilization noted for type II fatty acid synthase and other standalone ACP domains in the presence of divalent cations, which likely occurs due to the neutralization of negatively charged surface patches^23–26^. The isoelectric points of mACPb (pI 6.5) and mH0ACPb (pI 6.2) are very similar, so here the stability enhancement must have a different cause. Since Leu13781, Leu13785, Leu13788, Leu13797 and Ile13870 are buried in the structure of mH0ACPb, the entropic benefit of relieving water molecules from the need to solvate exposed hydrophobic side-chains in the H0 helix and on the surface of the canonical ACP motif could be responsible for lowering the free energy of the H0-docked state.

Our survey of predicted secondary structure suggests that H0-ACP motifs could be common, especially in the chain-extension modules of type I *cis*-AT PKS systems, where the prevalence of the H0 extension is estimated to be 98% (Table 4). Since some N-terminally truncated constructs fold successfully (e.g. mACPb) while others do not (e.g. mACPa), findings from the literature that rely on experiments conducted with ACP fragments should be viewed with caution unless they are supported by evidence of well-defined tertiary structure. For example, *in vitro* experiments that aimed to compare the recognition of carrier protein domains from the six chain-extension modules of the 6DEB PKS may instead have reported primarily on construct stability, since none of the described ACP fragments would possess intact H0 regions if prepared using the published primers^27,28^. In this case, later CD and NMR studies were able to confirm that three of the truncated constructs (*ery*ACP2, *ery*ACP3 and *ery*ACP6) were highly structured^11,29,30^, but the same level of supporting information has to our knowledge not yet been provided for *ery*ACP1, *ery*ACP4 or *ery*ACP5.

If the H0 region truly forms a normal part of ACP structure *in situ* within type I *cis*-AT PKS chain-extension modules, an obvious implication is that the linker between the ACP and the preceding domain might be shorter than was previously expected, imposing strict limits on the ability of the ACP to shuttle acyl substrates within and between PKS modules. The reach of an unstructured protein chain can be modelled on the dimensions of a polyproline type 2 helix (3.1 Å per residue^31^): an 8-residue helix followed by a 4-residue turn could unravel into a linker segment with a maximum extension of 37 Å. Our bioinformatics survey highlighted a trend for predicted pre-H0 linker sequences to be longer in modules that possess sequential AT and ACP domains (25 residues, 77 Å) than those in modules with KR-ACP configurations (9 residues, 28 Å; Table 4). In a module that possesses a KR domain, the ACP should visit more active sites than if the KR was absent, so this observation about linker length could be regarded as counter-intuitive. However, cryo-electron microscopy studies of the fifth chain-extension module from the pikromycin PKS suggest that the KR domain may undergo an end-to-end flipping motion during each reaction cycle^32^. Choreography of this sort could allow a H0-ACP unit to service all of its client domains despite being attached to a KR domain by a relatively short tether. By contrast, the AT domain appears to be much less mobile, relocating by <10 Å during the reaction cycle^32^. Chain-extension modules with AT-ACP configurations may therefore need a longer linker for the ACP to interact successfully with AT and KS domains within the same module as well as acyl-group acceptor sites in the KS domains of the next.

A preliminary sequence analysis^2^ highlighted a resemblance between a region named “helix 0” and an LxxLL motif found in coactivator peptides that bind to the surface of the androgen receptor^33^, prompting speculation that this feature might create an interface for interactions between the ACP and client domains. This hypothesis is ingenious, but unlikely: in our structures of mH0ACPa and mH0ACPb, the proposed LxxLL segment maps onto the middle of H1, the first long helix, with the leucine-equivalent side-chains contributing to the hydrophobic core (Supplementary Fig. S11B). Instead, we have shown that a LAARLAGL sequence further upstream can assemble an element of secondary structure that we call H0, which tucks in to form an integral part of an N-terminally extended ACP fold. Frequency analysis reveals that five positions in this region (LxxRLAxL) exhibit a degree of conservation similar to those of other key components of type I *cis*-AT PKS ACP domains (Supplementary Fig. S12A). Interestingly, alternative elaborations on the classic ACP fold are known, such as the carrier protein region of *S. cerevisiae* type I fatty acid synthase, which possesses two subdomains: an N-terminal section with a canonical ACP fold, followed by a 4-helix bundle “structural domain”^34^. The structural domain appears to have no direct involvement in substrate delivery, but rather maintains the stability of the canonical ACP domain and perhaps also contributes to interactions with architectural features of the fungal PKS assembly, such as the central wheel^35^.

As yet, no special role for the H0 region is apparent, apart from its ability to enhance the stability of the canonical ACP portions of mH0ACPa and mH0ACPb. Heterologous co-expression with Sfp produced both mH0ACPa and mH0ACPb in the *holo* form, so the H0 region appears not to disrupt intermolecular interactions with this *trans*-acting phosphopantetheinyl transferase enzyme. Although it has been shown to recognize specific residues along the length of H2, for example in a truncated *ery*ACP6 construct^30^, Sfp is also promiscuous enough to modify short unstructured peptides that possess the correct recognition sequence^36^. Successful modification by Sfp can therefore be interpreted as evidence that an ACP domain displays necessary sequence features, but is not sufficient to prove that it possesses native tertiary structure. Using ITC to define the affinities of mACPb and mH0ACPb for mKRb is perhaps a more rigorous test, as interactions of this sort would occur within an intact PKS module during a reductive reaction cycle. The additional N-terminal helix docks onto the face of the canonical ACP motif that is opposite to the modifiable serine residue (Fig. 2), so the minimal effect that the presence or absence of a H0 region has on the affinity of *apo* or *holo* species for KRb (Table 3) suggests that this part of the domain does not contribute the protein-protein interface. Further, the similar *K*_D_ values measured for *apo* forms of mACPb, mH0ACPb and mH0ACPa (Table 3) imply that all three species share a common interface region. Ketoreductase domains typically show much higher catalytic efficiency for acyl-ACP substrates than for acyl groups attached to pantetheine or CoA handles^37,38^, indicating that protein-protein recognition must play a significant role in the catalytic mechanism. Despite this, most KR domains from the 6DEB PKS system display little specificity for cognate acyl-ACP substrates over those involving non-cognate ACP domains, consistent with only highly conserved residues contributing to the interface, such as the DSL sequence that defines the Ppant modification site (mH0ACPa residues 11140–11142)^38,39^.

Site directed mutagenesis studies supported by docking simulations have demonstrated^40^: that ACP residues in the loop prior to H2 are crucial for KS-mediated chain-extension reactions^29^; that positions in H1 guide the translocation of the acyl chain onto a KS domain in the subsequent module^41^; that sites from the C-terminus of H1 through to the start of H2 govern AT-mediated interactions involved in the recruitment of new extender units^42^; and that regions close to the modifiable serine and in the loop prior to H3 promote the release of substrate chains by a terminal TE domain^43^ (although the latter finding has been disputed^30^). Future experiments will determine whether these inter-domain contacts are affected by the presence of the extended H0-ACP motif, but the results presented here indicate that it would be prudent to repeat any docking simulations that relied on homology models based on the truncated *ery*ACP2 construct^11^ with new models derived from the solution structures of mH0ACPa or mH0ACPb.

Synthetic biologists are enthused by the prospect of engineering megasynthase systems to produce novel drug-like fine chemicals, commodity chemicals and biofuels, but acknowledge that to date most attempts to construct chimeric synthases have failed to generate the expected target molecules in high yield^44^. For modular type I PKS systems, the efficiency of domain-swapped assembly-lines could probably be optimized if domain boundaries were defined more accurately and if detailed quantitative studies of inter-domain interactions were performed^45,46^. The work described in this article shows how such information can be obtained for acyl carrier protein domains.

## Materials and Methods

### Protein expression and purification

The sequence coding for mKRb from module 7 of the *Mycobacterium ulcerans* gene *MLSB* (Uniprot: Q32YM8; residues 13784–13874) was cloned into pVB, a modified pET28α vector in which the amino acid recognition sequence for thrombin had been replaced with that for tobacco etch virus (TEV) protease, preceded by an N-terminal His_6_-tag fused to GB1, the 56-residue B1 immunoglobulin binding domain of streptococcal protein G^47^. The pVB-mKRb plasmid was transformed into competent *E*. *coli* Tuner (DE3) cells (Merck) and the His_6_-GB1-mKRb fusion protein was expressed by growing the cells at 37 °C in 1 L of LB medium, prepared according to standard protocols^48^, with 30 μg/mL kanamycin (Sigma) for selection, to a 600 nm optical density of 0.8, followed by induction with 0.5 mM isopropyl β-D-1-thiogalactopyranoside (IPTG; Sigma) and incubation at 20 °C for 20 h. Proteolytic release and subsequent purification of the desired protein product were as described in Moretto *et al*.^9^.

The sequences coding for mACPa (Uniprot: Q6MZA4; residues 11098–11185; Table S2) and mH0ACPa (Uniprot: Q6MZA4; residues 11087–11185; Table S2) from module 5 of *MLSA1* were respectively cloned into pVH, a modified pET28α vector in which the recognition sequence for thrombin had been replaced with that for tobacco etch virus (TEV) protease. The pVH-mACPa and pVH-mH0ACPa plasmids were separately transformed into competent *E*. *coli* Tuner (DE3) cells (Merck). His_6_-mACPa and His_6_-mH0ACPa fusion proteins were expressed by growing the cells at 37 °C in 1 L of LB medium, prepared according to standard protocols^48^, with 30 μg/mL kanamycin (Sigma) for selection, to a 600 nm optical density of 0.8, followed by induction with 0.5 mM isopropyl β-D-1-thiogalactopyranoside (IPTG; Sigma) and incubation at 15 °C for 16 h. Cells were harvested by centrifugation (20 min; 3583 × g), resuspended in lysis buffer (50 mM Na_2_HPO_4_, 300 mM NaCl, pH 8.0) with 5 mM imidazole, 2.5 units/mL benzonase nuclease (EMD Millipore) and Sigmafast EDTA-free protease inhibitor cocktail (Sigma) and then lysed using an Emulsiflex C5 homogeniser (Glen Creston). Clarified lysates were passed through Ni-NTA resin (Qiagen), washed twice with lysis buffer containing 30 mM imidazole and eluted with lysis buffer containing 50 mM Na_2_HPO_4_, 300 mM NaCl, 300 mM imidazole and 0.01% (v/v) NaN_3_ at pH 8.0. The eluted proteins were exchanged into phosphate buffer (50 mM Na_2_HPO_4_, 150 mM NaCl, pH 7.5) and their His_6_-tags were cleaved by overnight incubation at 4 °C with TEV protease in 1 mL TEV buffer (1 M sodium phosphate, 10 mM EDTA, 20 mM dithiothreitol (DTT), pH 7.5). Released mACPa and mH0ACPa were further purified by size exclusion chromatography using an Äkta Purifier 10 system equipped with a Superdex 75 10/300 column (GE Healthcare) in phosphate buffer. The samples were concentrated using 5000 MWCO Vivaspin 20 columns (Sartorius Stedim). All expression and purification steps were monitored by SDS-PAGE (NuPAGE) 4–12% Bis-Tris gels (Life Technologies) stained with InstantBlue (Expedeon) (Fig. S2). The identity of each sample was confirmed by electrospray injection mass spectrometry (ESI-MS; PNAC facility, Department of Biochemistry, University of Cambridge; Table S3 and Fig. S3).

As described by Vance and coworkers^10^, the sequence for mACPb coded in module 7 of *MLSB* from *Mycobacterium ulcerans* (Uniprot: Q32YM8; residues 13784–13874) was cloned into a pET28α vector was for expression of N-terminally His_6_-tagged mACPb in *E*. *coli* Tuner (DE3) cells (Merck). The cells were grown at 37 °C in 1 L of LB medium, prepared according to standard protocols^48^, with 30 μg/mL kanamycin (Sigma) for selection, to a 600 nm optical density of 0.8, followed by induction with 0.5 mM isopropyl β-D-1-thiogalactopyranoside (IPTG; Sigma) and incubation at 15 °C for 16 h. The cells were harvested and mACPb was purified and concentrated as described above for mACPa, except His_6_-tag cleavage was performed using restriction grade thrombin (EMD Millipore). The sequence coding for mH0ACPb from module 7 of *MLSB* (Uniprot: Q32YM8; residues 13774–13874) was cloned into pVB for expression of His_6_-GB1-mH0ACPb in *E*. *coli* Tuner (DE3) cells (Merck) and was then cleaved and purified using the protocol described for mKRa above. For mACPb and mH0ACPb, all expression and purification steps were monitored by SDS-PAGE (NuPAGE) 4–12% Bis-Tris gels (Life Technologies) stained with InstantBlue (Expedeon) (Fig. S2) and the identity of the sample was confirmed by ESI-MS (PNAC facility, Department of Biochemistry, University of Cambridge; Table S3 and Fig. S3).

Uniformly ^15^N- and ^15^N/^13^C-labelled were prepared using M9 growth medium, prepared according to standard protocols^48^, supplemented with ^13^C_6_-D-glucose (Cambridge Isotope Laboratories) and/or ^15^N ammonium chloride, as required.

### Thermal denaturation experiments

For circular dichroism spectroscopy, protein samples were prepared at 0.2 mg/mL in phosphate buffer (45 mM Na_2_HPO_4_, 5 mM NaH_2_PO_4_, 150 mM NaF, 0.01% (v/v) NaN_3_, pH 7.5). Thermal denaturation profiles were obtained by monitoring the molar ellipticity ([θ]) at 220 nm on an Aviv Model 410 CD spectrometer. [θ] was recorded at 1 °C increments ranging from 20 °C to 95 °C. The unfolded state percentage was calculated using the formula F(*T*) = (([θ]_max_ − [θ]_*T*_)/([θ]_max_ − [θ]_min_)) × 100, where [θ]_max_ is the maximum observed value of [θ], [θ]_min_ is the minimum observed value and [θ]_T_ is the [θ] recorded at temperature, *T*. Melting temperatures *T*_m_ were estimated from the inflexion points of normalized melting curves. Reported *T*_m_ values are the mean of three technical replicates.

### Preparation of holo and acyl-loaded ACP samples

ACP constructs were prepared in the Ppant-attached *holo* form *in vivo* by co-expression. Two expression vectors, one coding for the ACP construct and the other for the broad specificity phosphopantetheinyl transferase Sfp (pET-Sfp)^49^, were co-transformed into *E. coli* Tuner(DE3) cells. The expression and purification procedures for *holo* ACP species were as described above for *apo* species. The modification state of the ACP domain was confirmed by ESI-MS (PNAC facility, Department of Biochemistry, University of Cambridge; Table S3 and Fig. S3).

Loading reactions for β-hydroxybutyryl-mH0ACPa were set up *in vitro*. An *apo* mH0ACPa sample (0.1 mM) was incubated at 27 °C for 2 h with Sfp (4.4 mM) and β-hydroxybutyryl-CoA (2 mM) in phosphate buffer (45 mM Na_2_HPO_4_, 5 mM NaH_2_PO_4_, 150 mM NaCl, 0.01% (v/v) NaN_3_, pH 7.5) supplemented with 10 mM MgCl_2_. To separate the loaded protein from Sfp and any excess substrate, the mixture was purified by size exclusion chromatography, as described above. The identity of the eluted protein was confirmed by ESI-MS (PNAC facility, Department of Biochemistry, University of Cambridge; Table S3 and Fig. S4).

### Isothermal titration calorimetry experiments

ITC measurements of affinity (*K*_D_), stoichiometry (*n*) and apparent enthalpy change (*ΔH°*) were obtained using a VP-ITC microcalorimeter (MicroCal Inc.). Samples for cell and injectant solutions were prepared in the same buffer (25 mM HEPES, 50 mM NaCl, 0.01% (v/v) NaN_3_, pH 7.5). All samples were degassed under vacuum using a ThermoVac accessory (MicroCal Inc.) and loaded into the cell and the syringe. All experiments were conducted at 30 °C with a stirring speed of 300 rpm and a sequence of 29 injections of 10 μL, each lasting 7.1 s, and with a 240 s interval between each injection. The cell volume was 1400 μL. The first injection was set to a smaller volume (2 μL in 3.3 s) to allow the cell and needle solutions to mix, and the resulting heat change was disregarded in the later analysis. Control experiments were run to confirm that dilution heat changes caused by the titration of injectant into the cell solution were negligible. Initial concentrations were: KRb in the cell, 50 μM; NADPH or NADP+ in the cell, 5 mM; and ACP species in the syringe, 1 mM. Dilution heat change thermograms for each cell sample were subtracted from the final trace before integration with respect to time to generate isotherm traces. All isotherm traces were analysed using ORIGIN, version 7.0 (MicroCal, Inc.) with a model that assumed a single binding site. Reported *K*_D_ values are the mean of three technical replicates. Representative ITC thermograms and isotherm plots for experiments with mKRb in the cell and various ACP species in the syringe are displayed in Fig. S6 and S7.

### NMR experiments for assignment and distance restraints

Samples for nuclear magnetic resonance (NMR) spectroscopy were prepared at concentrations of 200–800 μM in phosphate buffer supplemented with 10% D_2_O (Sigma) and 0.0025% 3,3,3-trimethylsilylpropionate (Sigma) in 5 mm Ultra-Imperial grade NMR tubes (Wilmad) to a final volume of 600 μL. 10 mM DTT was added to *holo* samples. All mH0ACPa samples were studied at 298 K and all mH0ACPb samples at 283 K. [^1^H,^15^N]-HSQC, ^15^N-TOCSY-HSQC, ^15^N-nuclear Overhauser effect spectroscopy (NOESY)-HSQC, ^13^C-NOESY-HSQC, HNCA, HNCOCA, HNCACB and CBCA(CO)NH spectra were recorded on a Bruker DRX500 spectrometer equipped with a *z*-shielded gradient triple resonance probe, using standard procedures^50^. For *apo* mH0ACPa, a ^13^C-NOESY-HSQC spectrum was collected on a Bruker Avance DRX800 spectrometer equipped with a 5 mm TXI CryoProbe. All NMR spectra were processed using the Azara package (www.ccpn.ac.uk/azara), then analysed and assigned using CcpNmr Analysis software^51^. To compare resonance positions in [^1^H,^15^N]-HSQC spectra of different ACP species, average chemical shift differences were determined using the formula

$${\rm{\Delta }}{\delta }_{{\rm{a}}{\rm{v}}}={\{0.5{({\rm{\Delta }}{\delta }_{{\rm{H}}})}^{2}+0.1{({\rm{\Delta }}{\delta }_{{\rm{N}}})}^{2}\}}^{0.5}$$

Residual dipolar coupling (RDC) contributions to ^1^*J*^NH^ values were measured using ^15^N-labeled samples of mH0ACPa and mH0ACPb that were partially aligned by addition of filamentous phage Pf1 (Profos AG, Regensburg, Germany) to a final phage concentration of 15.0 mg mL^−1^ (yielding a ^2^H splitting of 10 Hz). Independent IPAP-[^1^H,^15^N]-HSQC datasets^52^ were collected in the presence and absence of phage. The PALES program^53^ was used to estimate initial magnitudes for the axial and rhombic components of the alignment tensor from preliminary structures determined without RDC restraints.

### Determination of solution structures for apo mH0ACPa and mH0ACPb

Structures of *apo* mH0ACPa and mH0ACPb were calculated from extended templates by simulated annealing using ARIA 2.3^54^, with manual screening of ambiguous restraints. Backbone ϕ and ψ dihedral angle restraints were determined from chemical shifts using the DANGLE program^55^. NOE distance restraints generated by the resonance assignment process and dihedral angle restraints were fed as input. RDC restraints were incorporated into the structure calculations via the SANI potential in square-well mode. Nine iterations were performed, each using 20 structures, except for the final round, in which 100 were calculated, followed by refinement in explicit solvent for the 20 lowest energy structures, all of which were selected for the final ensemble, which in each case contained no distance violations >0.5 Å and no dihedral angle restraint violations >5°. The atomic coordinates of the final ensembles for *apo* mH0ACPa and mH0ACPb were deposited in the Protein Data Bank under ID codes 6H0J and 6H0Q, respectively; corresponding NMR resonance assignments were deposited in the Biological Magnetic Resonance Data Bank under accession codes 34299 and 34301. The structure statistics for Table 1 were determined using the PSVS server^56^.

### ^15^N nuclear spin relaxation experiments

^15^N nuclear spin relaxation experiments were recorded using standard procedures^50^ on a Bruker DRX500 spectrometer, at 298 K for mH0ACPa and at 283 K for mH0ACPb.^15^N *T*_1_ delays (ms): 10, 50, 100, 150, 250, 400, 550, 700, 850, 1000. ^15^N *T*_2_ delays (ms): 14.4, 28.8, 43.2, 57.6, 72.0, 86.4, 100.8, 155.2. The heteronuclear NOE reference and saturation experiments were carried out in duplicate to allow an estimation of the error. Relaxation parameters were analysed using the ROTDIF-1.1 package^57^.

### Bioinformatics

Optimal alignments of ACP structures were determined using the frTM-align program^58^. All protein structure figures were prepared using PyMOL (https://pymol.sourceforge.net/). The amino acid sequences of 770 carrier protein domains together with 100 residues of preceding sequence were excised from the NRPS-PKS database^59^ and then submitted to the SSpro component of the Scratch protein prediction server at https://www.ics.uci.edu/baldig/scratch/^17^ for three-state secondary structure prediction. Multiple sequence alignments were obtained using the MUSCLE server at https://www.ebi.ac.uk/Tools/msa/muscle/^60^. Graphical representations of amino acid frequencies in these alignments were generated using the WebLogo server at https://weblogo.berkeley.edu/^61^. Evolutionary covariance analysis was performed using the GREMLIN server at https://gremlin2.bakerlab.org/^62^.

## Supplementary information

Supplementary Information
