# Supplementary material for: Modular type I polyketide synthase acyl carrier protein domains share a common N-terminally extended fold
Source: Sci Rep. 2019 Feb 20;9:2325. doi: 10.1038/s41598-019-38747-9 (PMC6382882; doi:10.1038/s41598-019-38747-9)
Supplement: Supplementary file 1 — Supplementary Information [file 41598_2019_38747_MOESM1_ESM.pdf]

Supplementary Information

TITLE
Modular type I polyketide synthase acyl carrier protein domains share a common N-terminally extended fold.

AUTHORS
Luisa Moretto[1], Rachel Heylen[2], Natalie Holroyd[3], Steven Vance[4] & R. William Broadhurst[2]*

AFFILIATIONS
[1] Department of Chemistry and Biomedical Sciences, Linnaeus University, Smålandsgatan-24, 392 34 Kalmar, Sweden.
[2] Department of Biochemistry, University of Cambridge, 80 Tennis Court Road, Cambridge CB2 1GA, U.K.
[3] Department of Medical Physics and Bioengineering, University College London, London WC1E 6BT, U.K.
[4] Crescendo Biologics Ltd, Meditrina Building 260, Babraham Research Campus, Cambridge CB22 3AT, U.K.
* Correspondence and requests for materials should be addressed to R.W.B. (email: rwb1002@cam.ac.uk)

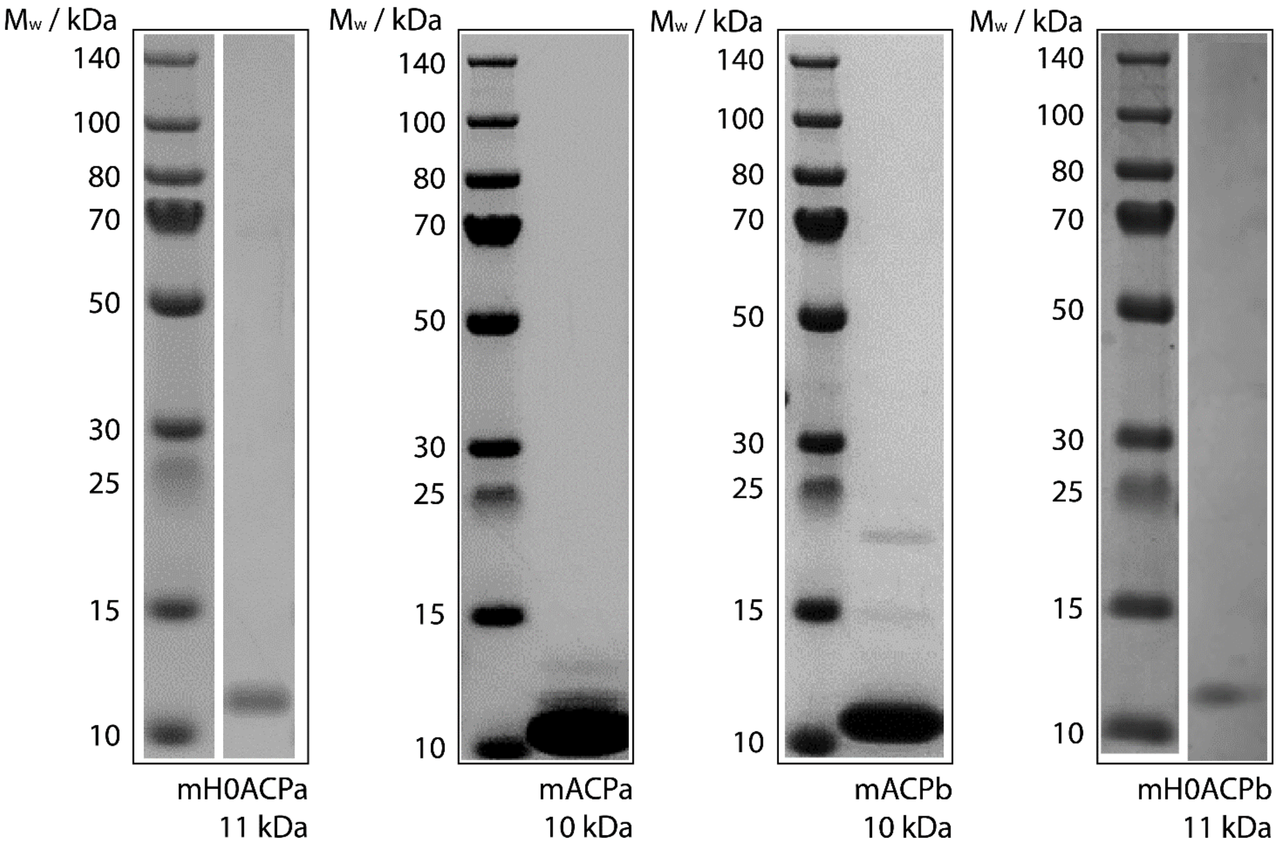

Supplementary Figure S1

Each panel displays a 4-12 % Bis-Tris gel containing Thermo Scientific PageRuler prestrained protein ladder (left lane) and the indicated protein construct after purification by size exclusion chromatography (right lane).

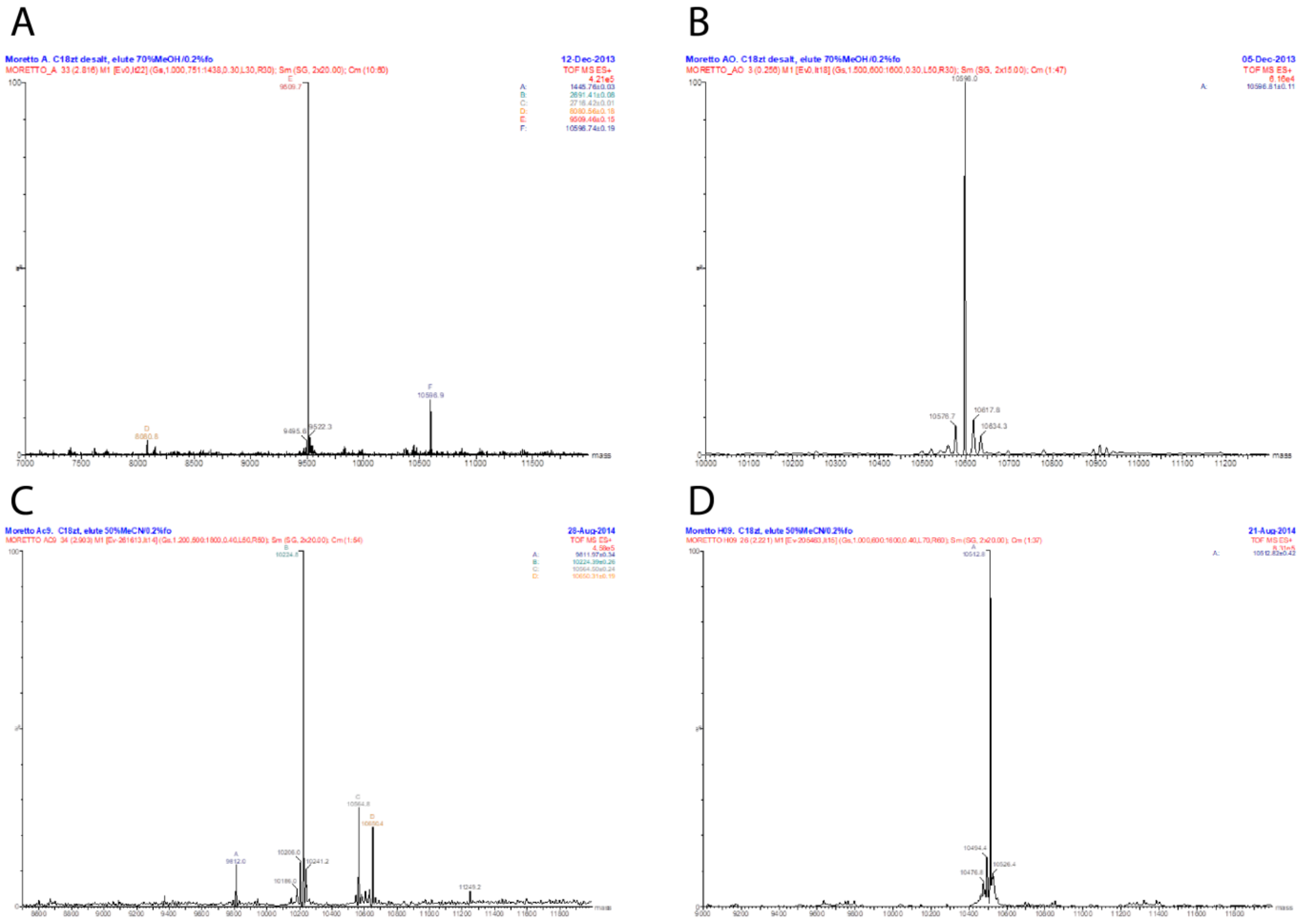

Supplementary Figure S2

Deconvoluted electrospray mass spectra for samples of: (A) *apo* mACPa (9509.7 Da); (B) *apo* mH0ACPa (10598.0 Da); (C) *apo* mACPb (10224.5 Da); and (D) *apo* mH0ACPb (10512.8 Da).

a

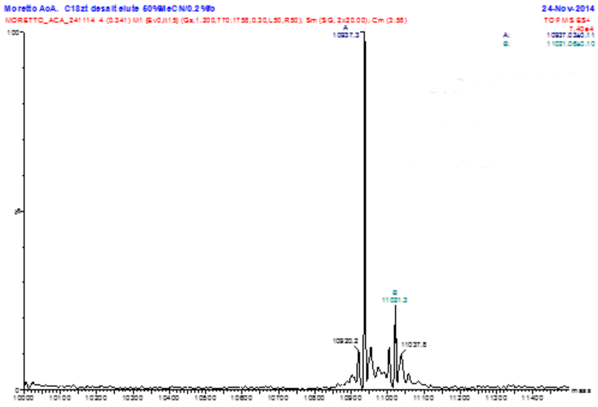

b

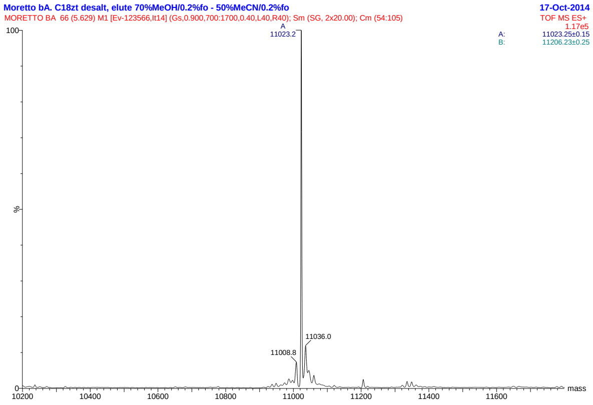

Supplementary Figure S3

Deconvoluted electrospray mass spectra for samples of: (a) *holo* mH0ACPa (10937.1 Da); and (b) β-hydroxybutyryl-mH0ACPa (11023.1 Da).

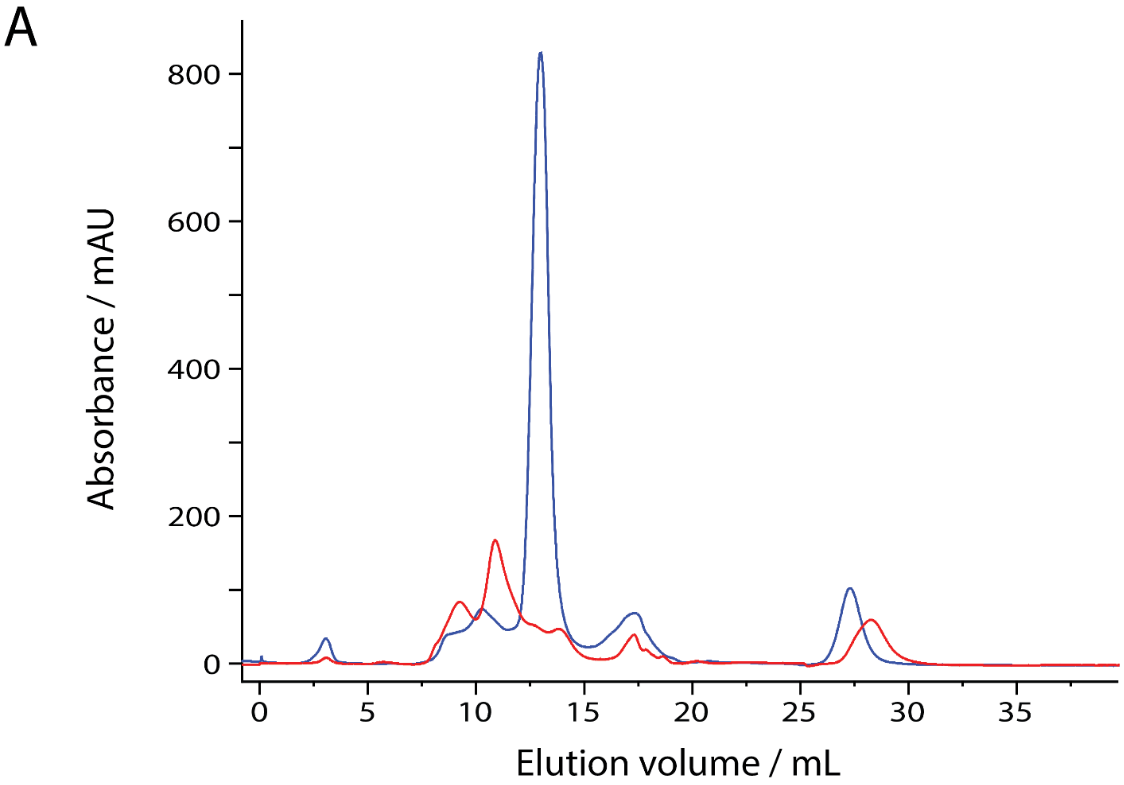

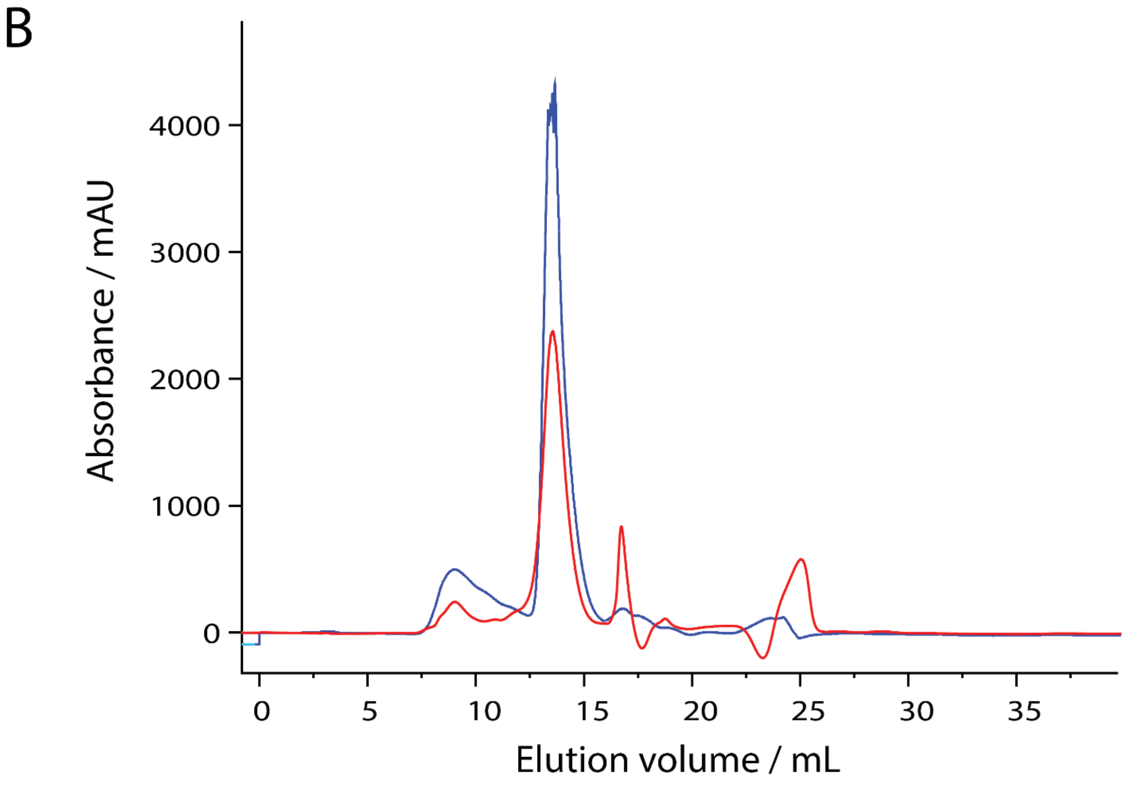

Supplementary Figure S4

Size exclusion chromatography elution profiles for: **(A)** mACPa (red) and mH0ACPa (blue); and **(B)** mACP9 (red) and mH0ACP9 (blue).

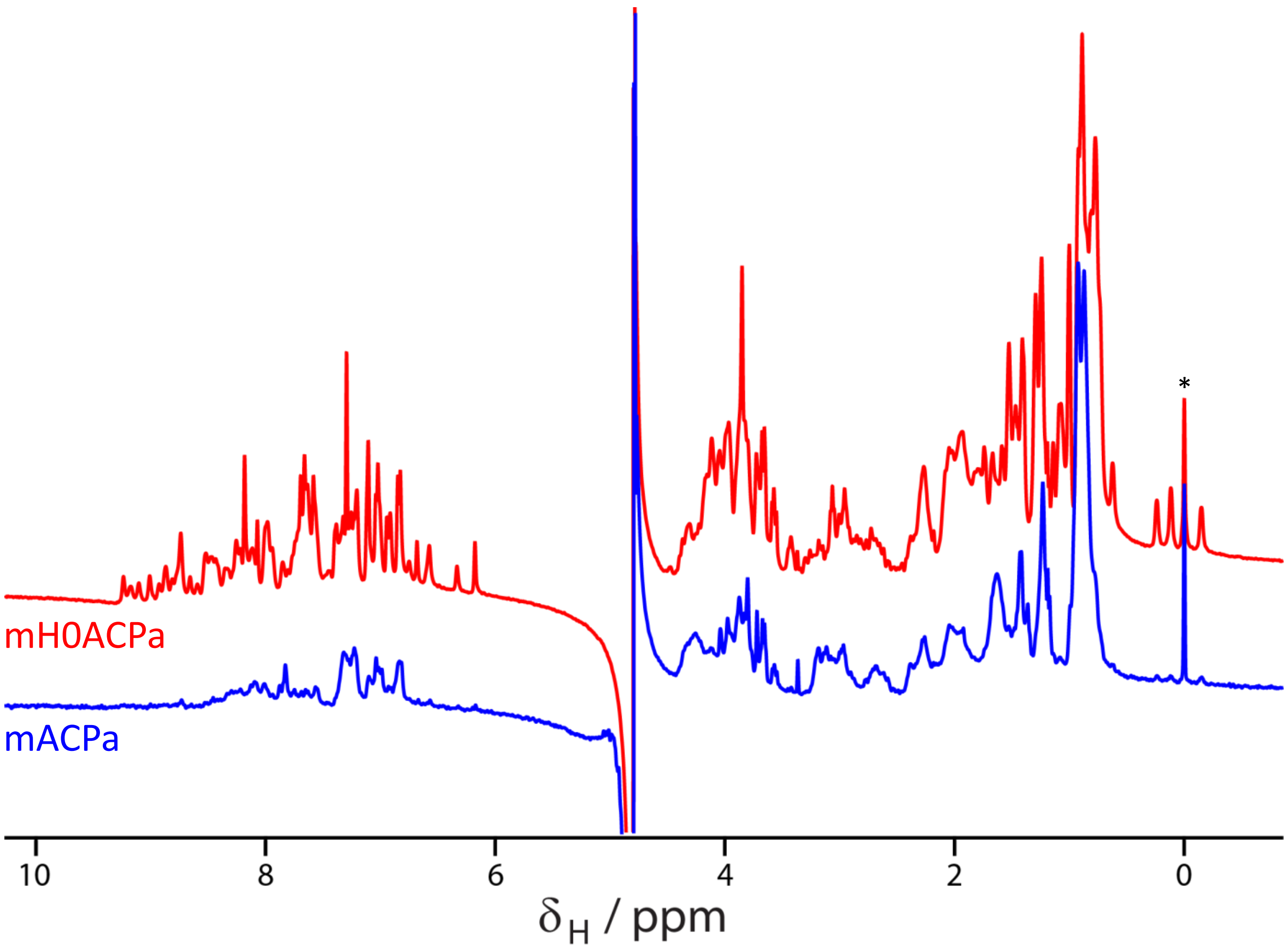

Supplementary Figure S5

1D ^{1}H spectra of *apo* mACPa (blue) and *apo* mH0ACPa (red). *, reference signal from 3,3,3-trimethylsilylpropionate.

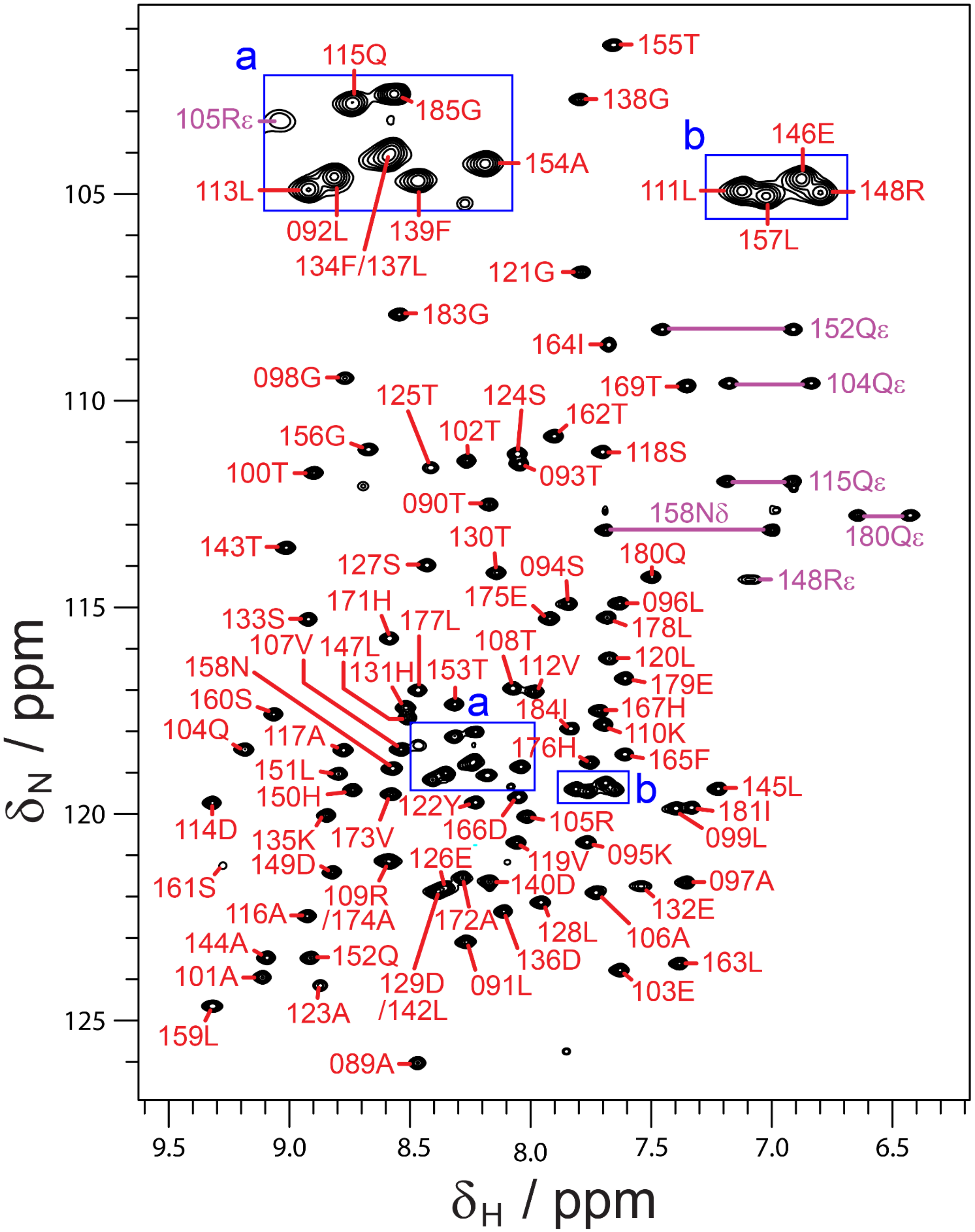

Supplementary Figure S6

[^{1}H,^{15}N]-HSQC spectrum of *apo* mH0ACPa, showing residue assignments for backbone amide sites. Pairs of resonances from side-chain amide sites are connected using magenta lines. Assignments for closely spaced signals are displayed in two inset panels. Add 11000 to obtain the sequence position within MLSA1.

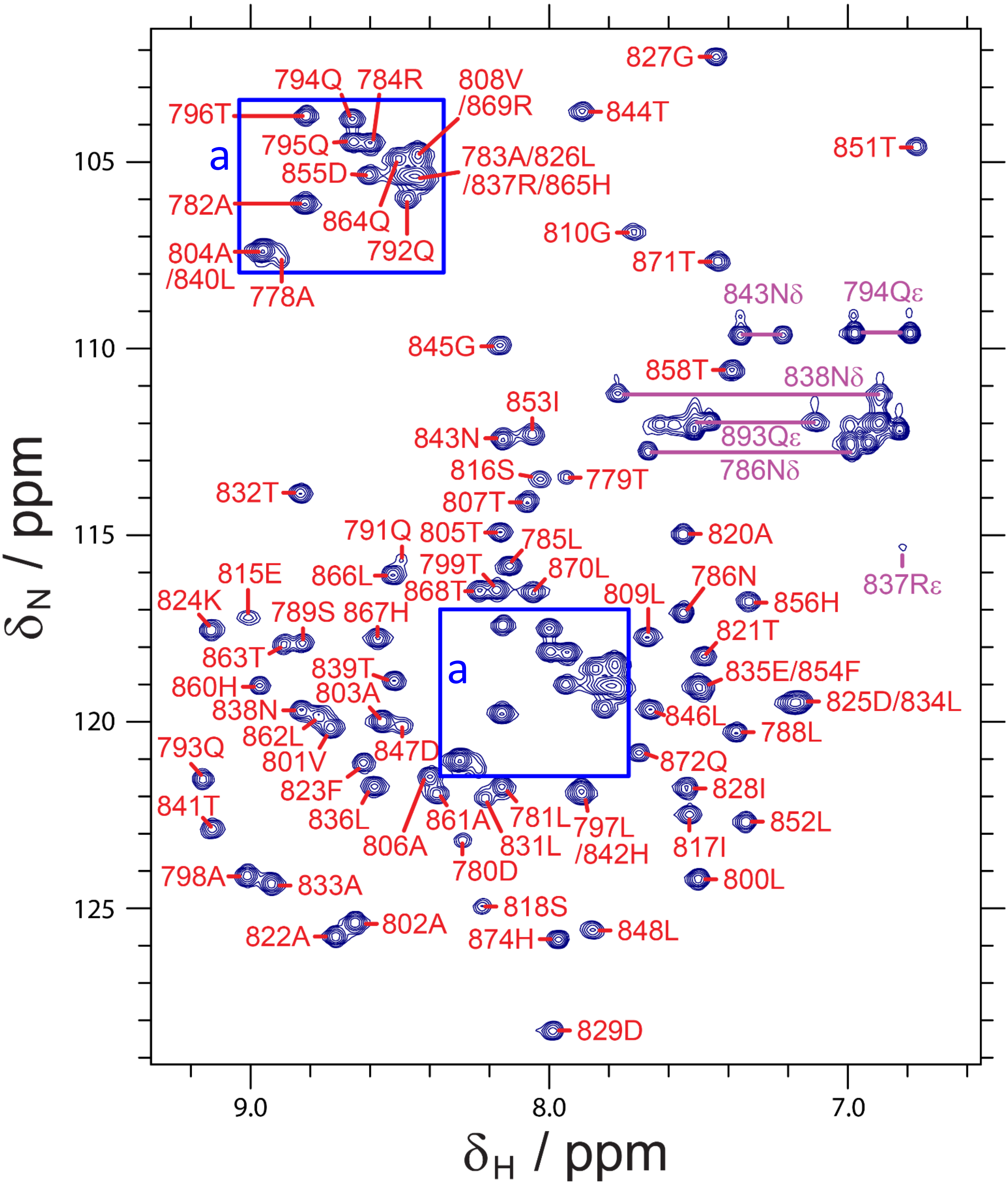

Supplementary Figure S7

[^{1}H,^{15}N]-HSQC spectrum of *apo* mH0ACPb, showing residue assignments for backbone amide sites. Pairs of resonances from side-chain amide sites are connected using magenta lines. Assignments for closely spaced signals are displayed in the inset panel. Add 13000 to obtain the sequence position within MLSB.

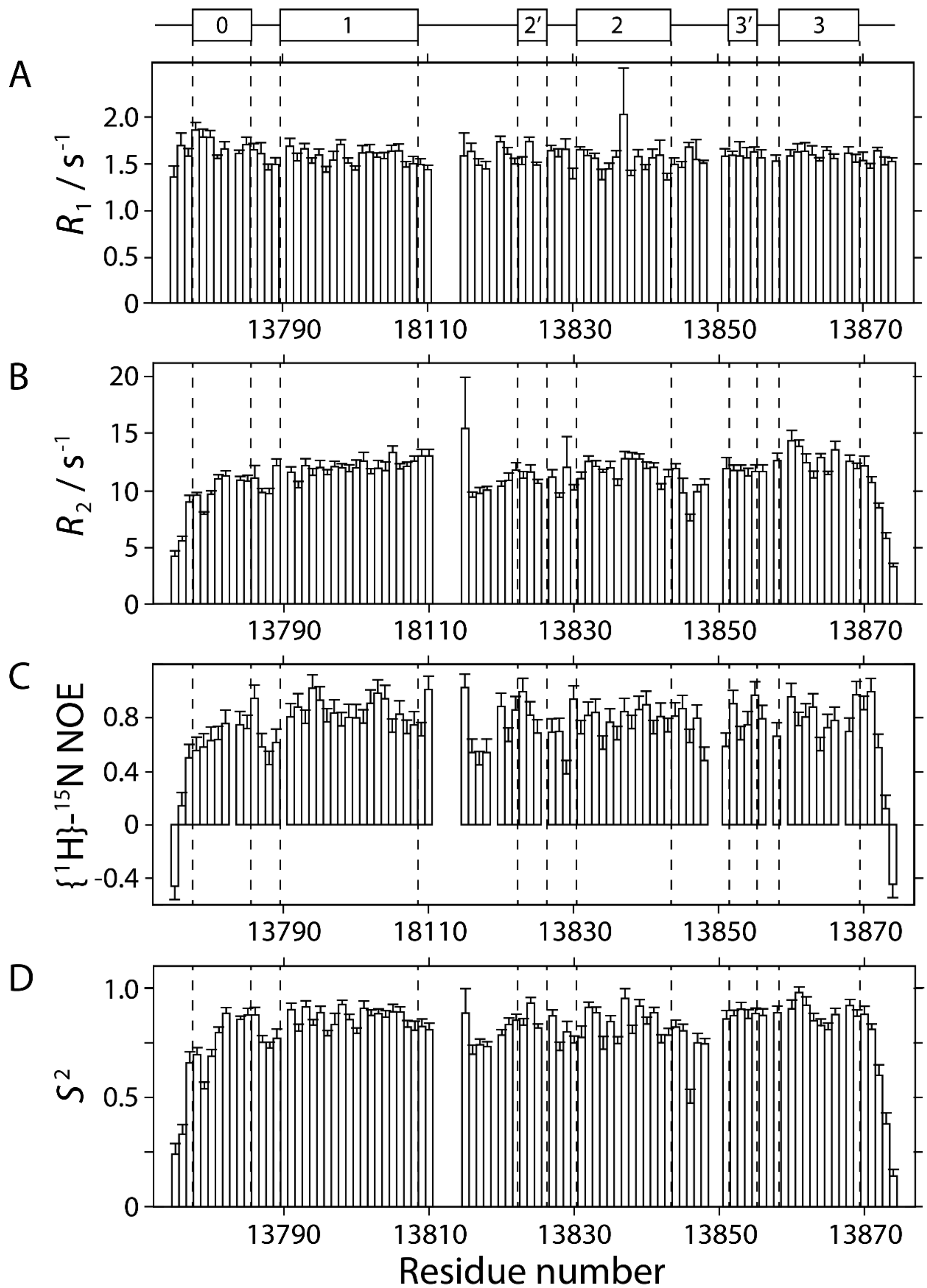

Supplementary Figure S8

Underneath a schematic defining the boundaries of the secondary structure elements in *apo* mH0ACPb, nuclear spin relaxation parameters for backbone amide sites are plotted as a function of residue number for: (A) the ^{15}N longitudinal relaxation rate, R_1; (B) the ^{15}N transverse relaxation rate, R_2; (C) the $\{^1H\}$-^{15}N nuclear Overhauser effect ratio (I'/I_0, where I' is the intensity when the ^1H spectrum has been saturated and I_0 is the intensity in the reference spectrum); and (D) the Lipari-Szabo the order parameter, S^2.

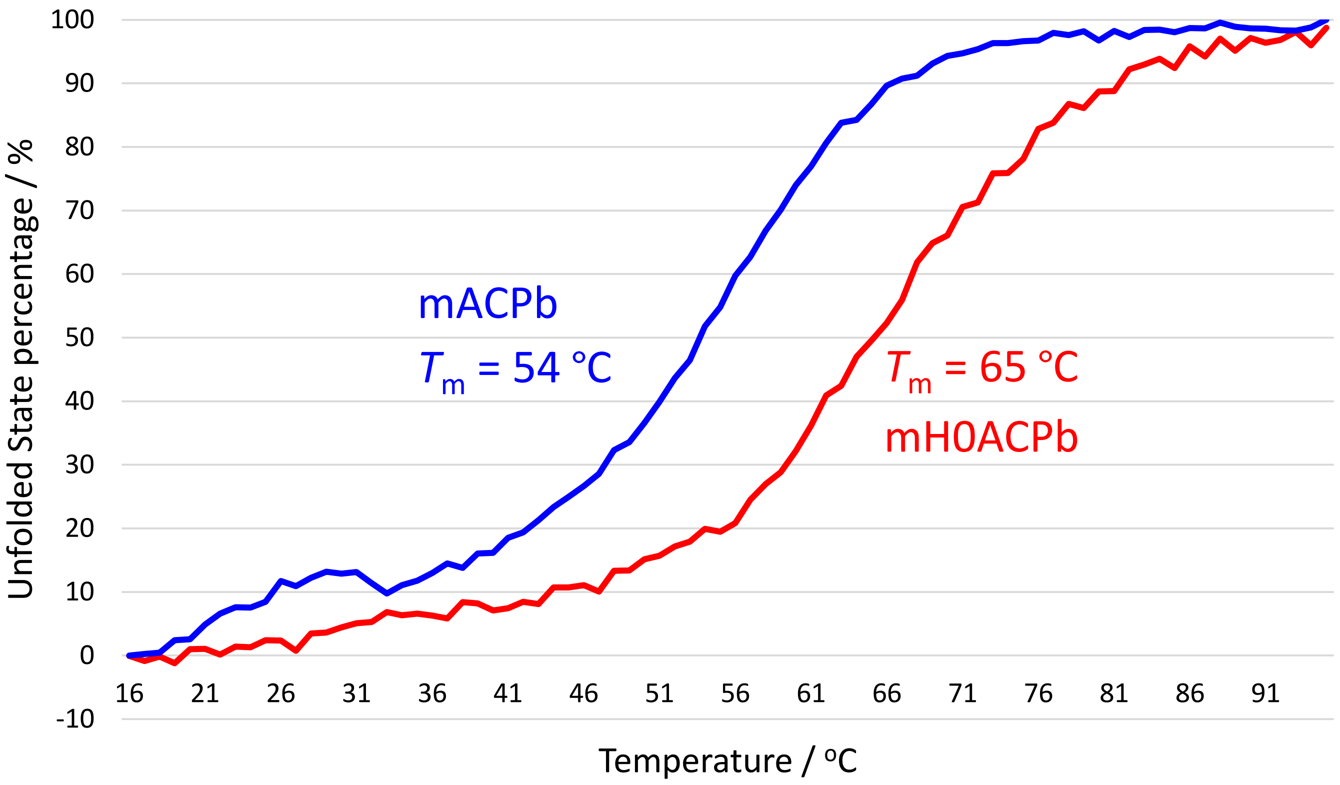

Supplementary Figure S9

Representative circular dichroism thermal denaturation curves for *apo* mACPb (blue) and *apo* mH0ACPb (red), obtained by following the mean residue ellipticity at 222 nm.

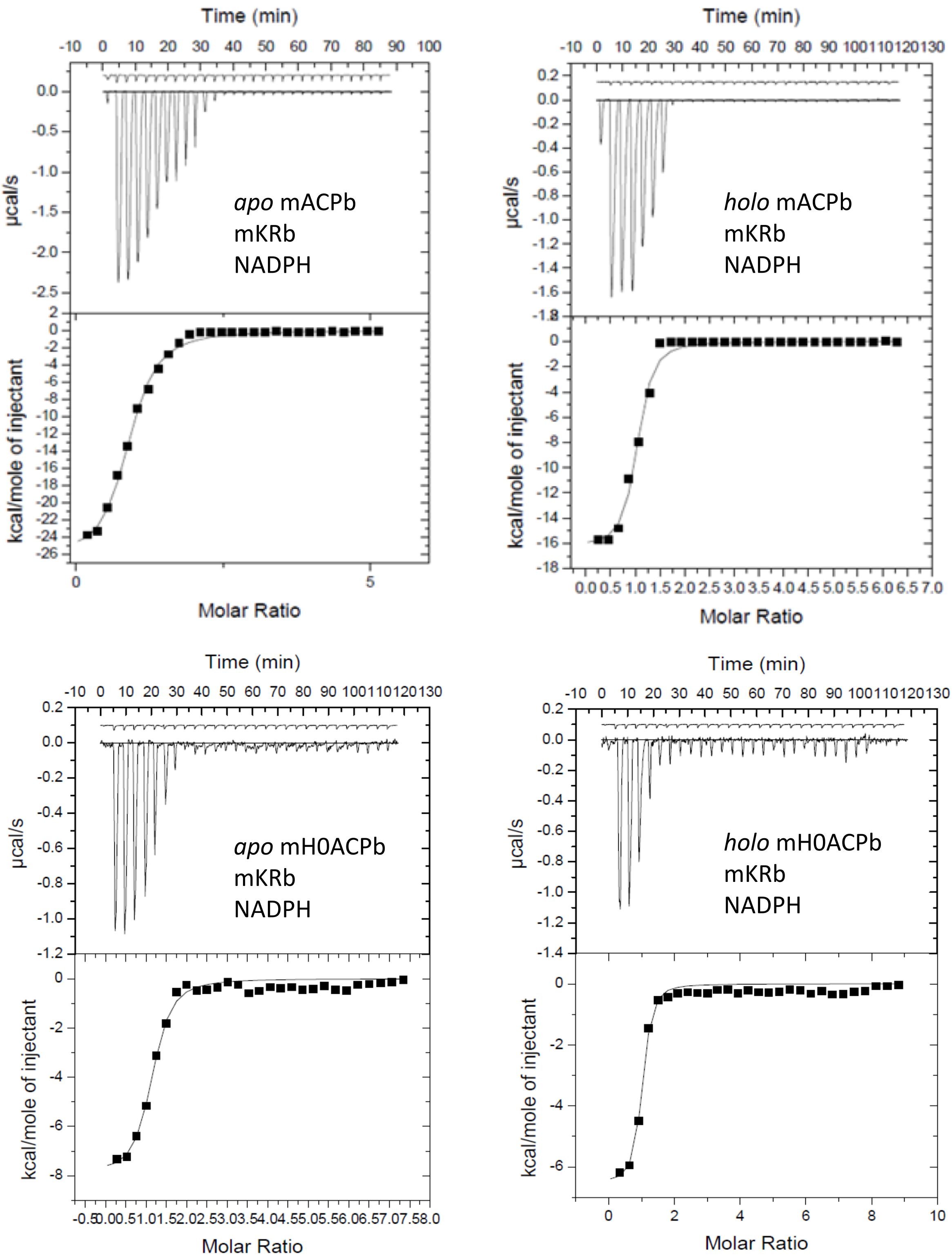

Supplementary Figure S10

Representative ITC thermograms (upper panels) and isotherm plots (lower panels), all with mKRb and NADPH in the cell and showing consecutive injections: (A) *apo* mACPb; (B) *holo* mACPb; (C) *apo* mH0ACPb; and (D) *holo* mH0ACPb. Thermogram traces for dilution control experiments are shown at the top of each upper panel.

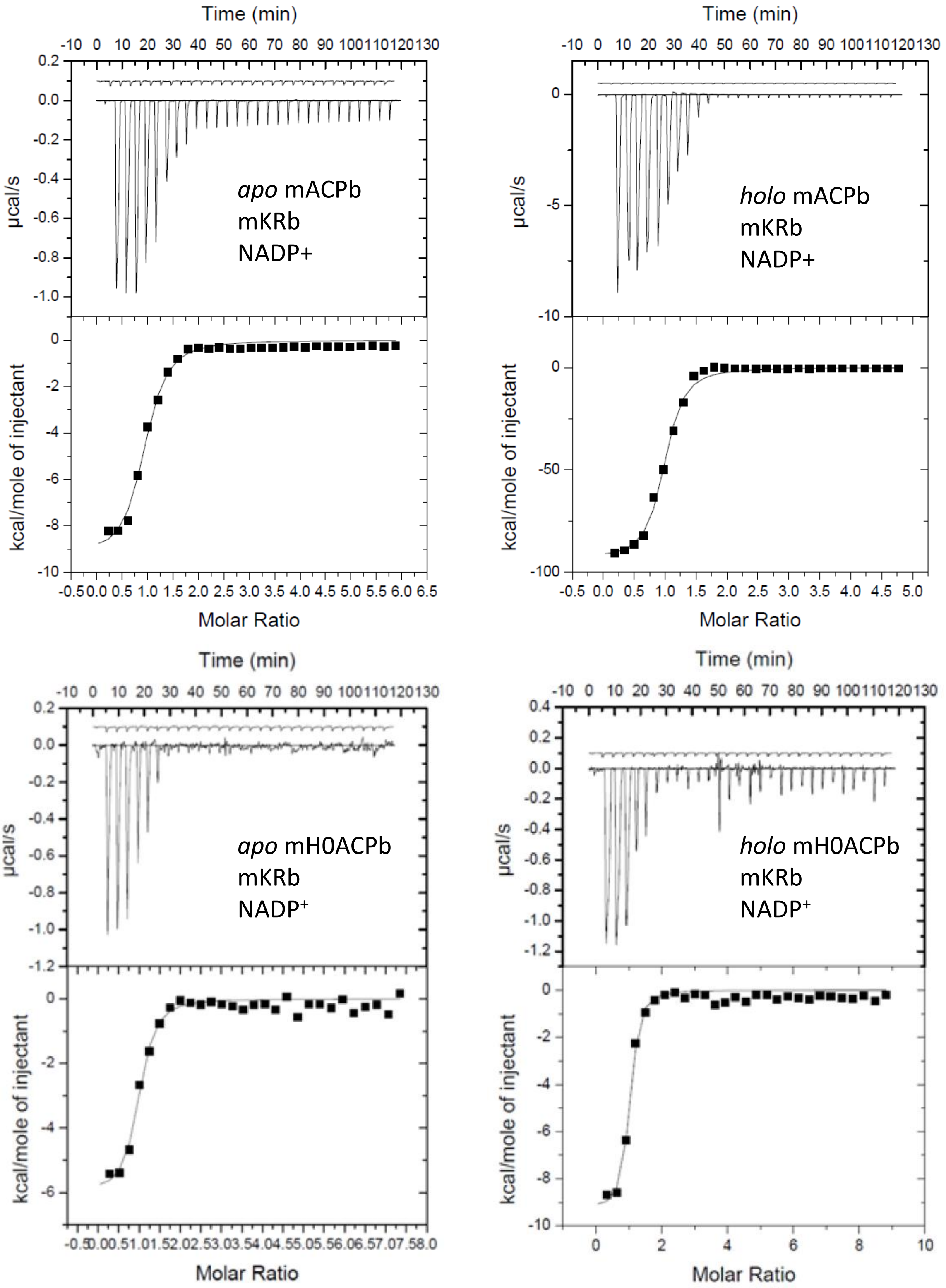

Supplementary Figure S11

Representative ITC thermograms (upper panels) and isotherm plots (lower panels), all with mKRb and $NADP^+$ in the cell and showing consecutive injections: (A) *apo* mACPb; (B) *holo* mACPb; (C) *apo* mH0ACPb; and (D) *holo* mH0ACPb. Thermogram traces for dilution control experiments are shown at the top of each upper panel.

A

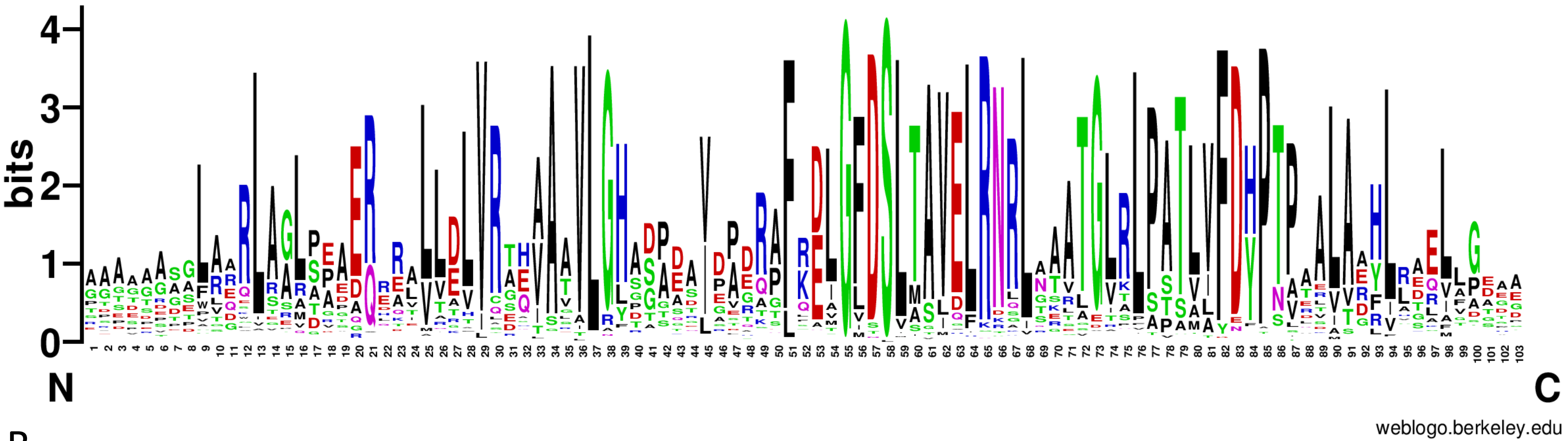

B

```
                  "helix 0"                      canonical ACP domain
Os11 DTGGGLDGLAGLPEDEQRARVMELVRRQVAAVLVVD-PDDVGPDTGFVELGLDSLTAVELRNRLGRATGLRLPVTLVFDHPSPGALAGYLRQRLTTA
Spn2 DAESLRKRLGRLPDAEQHRILLDLVRMHVAAVLGFAGSQEITADGTFKVLGFDSLTVVELRNRINGATGLRLPATLVFNYPTPDALAAHLVTALSAD
Am16 ARAGLAERLAVLPEEQRLPFVVDLVRAEAATVLGHGSADAVDARREFRGLGFDSLTAIELRNRLGKASGLTLTATLVFDYPTPQQLAEHLLDELLGA
Ery4 AGPNVVDRLAGRSESDQVAGLAELVRSHAAAVSGYGSADQLPERKAFKDLGFDSLAAVELRNRLGTATGVRLPSTLVFDHPTPLAVAEHLRDRLFAA
mH0a TATLLTSKLAGLTATEQRAVTRKLVLDQAASVLGYASTESLDTHESFKDLGFDSLTALELRDHLQTATGLNLSSTLIFDHPTPHAVAEHLLEQIPGI
mH0b AATDLAARLNGLSPQQQQQTLATLVAAATATVLGHHTPESISPATAFKDLGIDSLTALELRNTLTHNTGLDLPPTLIFDHPTPHALTQHLHTRLTQS
        H0              H1                        H2′          H2              H3′         H3
```

Supplementary Figure S12

(A) Sequence logo plot for an alignment of 349 ACP domains from modular type I polyketide synthase systems. (B) Sequence alignment of ACP domains displaying the domain definitions suggested by Keatinge-Clay (2012) labelled at the top and in underlined italics, secondary structure predictions of helices from SSPRO (cyan), experimental heliical regions (magenta), and secondary structure annotation for the H0-ACP fold at the bottom. ACP domain sources: Os11, module 11 of oxazolomycin PKS; Spn2, module 2 of the spinosyn PKS; Am16, module 16 of the amphotericin PKS; Ery4, module 4 of the 6DEB PKS; mH0a, module 5 of MLSA1; mH0b, module 7 of MLSB. Keatinge-Clay, A.T. (2012) *Nat. Prod. Rep.*, **29**, 1050-1073.

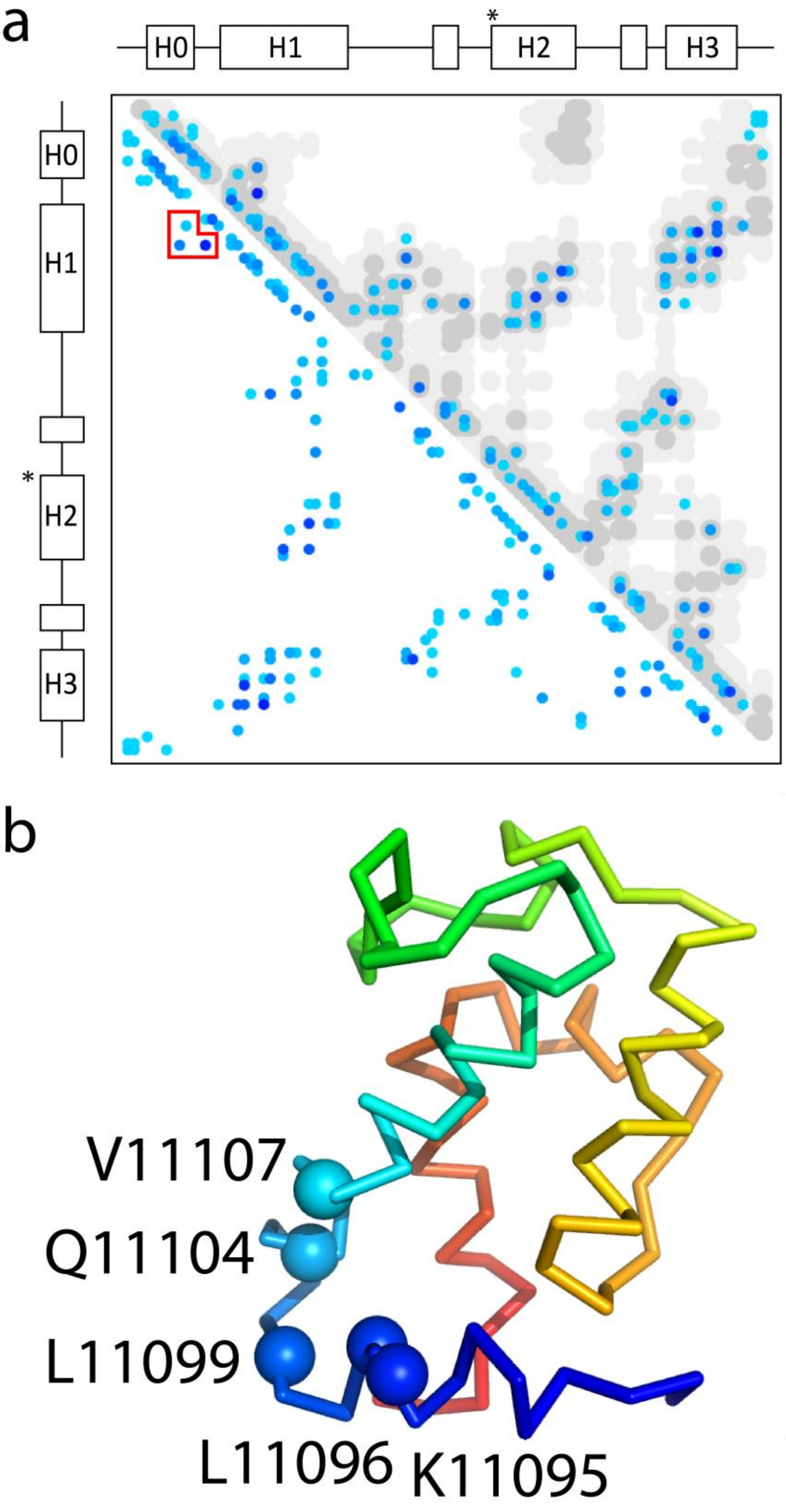

Supplementary Figure S13

(a) Contact map for mH0ACPa (top right) and contacts predicted using evolutionary covariance analysis (bottom left; contacts across the H0-H1 hinge are in the red box). (b) Location of backbone amide N atoms of residues identified in the H0-H1 hinge identified by GREMLIN.

A mACPa

```
GGLTATEQRA VTRKLVLDQA ASVLGYASTE SLDTHESFKD LGFDSLTALE LRDHLQTATG
LNLSSTLIFD HPTPHAVAEH LLEQIPGIG
```

B mH0ACPa

```
GSTATLLTSK LAGLTATEQR AVTRKLVLDQ AASVLGYAST ESLDTHESFK DLGFDSLTAL
ELRDHLQTAT GLNLSSTLIF DHPTPHAVAE HLLEQIPGIG
```

C mACPb

```
GSHMRLNGLS PQQQQQTLAT LVAAATATVL GHHTPESISP ATAFKDLGID SLTALELRNT
LTHNTGLDLP PTLIFDHPTP HALTQHLHTR LTQSH
```

D mH0ACPb

```
GAASAATDLA ARLNGLSPQQ QQQTLATLVA AATATVLGHH TPESISPATA FKDLGIDSLT
ALELRNTLTH NTGLDLPPTL IFDHPTPHAL TQHLHTRLTQ SH
```

E mKRb

```
GDSLITRPLT TATGSAPATT AAGLLHLSWP PHPDTTTDTD TDTDALRYQV IAEPTQQLPR
YLHDLHTSTD LHTSTTEADV VVWPVPVPSN EELQAHQASD TAVSSRIHTL TRQTLTVVQD
WLTHPDTTGT RLVIVTRHGV STSAHDPVPD LAHAAVWGLI RSAQNEHPGR FTLLDTDDNT
NSDTLTTALT LPTRENQLAI RRDTIHIPRL TRHSSDGALT APVVVDPEGT VLITGGTGTL
GALFAEHLVS AHGVRHLLLT SRRGPQAHGA TDLQQRLTDL GAHVTITACD ISDPEALAAL
VNSVPTQHRL TAVVHTAAVL ADTPVTELTG DQLDQVLAPK IDAAWQLHQL TYEHNLSAFI
MFSSMAGMIG SPGQGNYAAA NTALDALADY RHRLGLPATS LAWGYWQTHT GLTAHLTDVD
LARMTRLGLM PIATSHGLAL FDAALATGQP VSIPAPINTH TLARHARDNT LAPILSALIT
TPRRR
```

Supplementary Figure S14

Amino acid sequences are shown for the following protein fragments: mACPa (A), mH0ACPa (B), mACPb (C), mH0ACPb (D) and mKRb (E).
